# Mechanistic Insights Into Qidan Yixin Decoction for Diabetic Cardiomyopathy via Macrophage Polarization

**DOI:** 10.1155/jdr/7578626

**Published:** 2025-07-31

**Authors:** Yi Liu, Juan Zhang, Wei Gao, Quancheng Han, Yitao Xue, Xiujuan Liu

**Affiliations:** ^1^The First Clinical Medical College, Shandong University of Traditional Chinese Medicine, Jinan, China; ^2^Department of Cardiology, Shandong University of Traditional Chinese Medicine Affiliated Hospital, Jinan, China

**Keywords:** diabetic cardiomyopathy, experimental validation, machine learning, macrophage polarization, network pharmacology

## Abstract

**Background:** Diabetic cardiomyopathy (DCM) has a multifactorial etiology, and no specific treatment is available for its management. Qidan Yixin decoction (QDYXD) demonstrated encouraging clinical results in treating DCM; however, its underlying mechanics are yet unclear.

**Methods:** Network pharmacology was applied to determine the active ingredients and targets of QDYXD. The GeneCards and GEO databases were used to retrieve genes associated with DCM and macrophage polarization. These targets were subjected to GO and KEGG enrichment analyses, immune infiltration study, and PPI network design. The core targets were further refined using SVM-RFE, LASSO, and random forest algorithms. Docking the core targets with the main active components followed. For experimental validation, rat models were created.

**Results:** There were 48 potential targets in all. Quercetin, methyl palmitate, luteolin, and tanshinone IIA were identified as the primary active components. Enrichment analysis indicated that one of the key pathways associated with the potential targets was the signaling pathway of HIF-1. Machine learning techniques were used to identify two core targets, LDHA and PGK1. Animal experiments demonstrated that QDYXD can suppress the upregulation of PGK1, LDHA, and HIF1A; block the polarization of M1 macrophages; and considerably enhance DCM rats' cardiac function.

**Conclusion:** QDYXD improves cardiac function in DCM by attenuating M1 macrophage polarization and inhibiting the HIF-1 signaling pathway, specifically through the modulation of PGK1, LDHA, and HIF1A. This study provides preliminary insights into how QDYXD modulates macrophage polarization during DCM treatment. Moreover, the identification of potential active components and core molecular targets of QDYXD offers promising directions for future drug development in DCM therapy.

## 1. Introduction

Diabetic cardiomyopathy (DCM) is a cardiac condition marked by structural and functional abnormalities of the myocardium [1]. DCM is regarded as a primary cause of heart failure in diabetic people and is one of the main cardiovascular consequences of diabetes [2]. About 35% of those with Type 2 diabetes will experience cardiac dysfunction, and there is a known link between the risk of heart failure and high glycated hemoglobin (HbA1c) levels [3]. In DCM, chronic inflammation is acknowledged as a major pathogenic component, with increased macrophage infiltration serving as a primary contributor [4]. Recent data points to macrophage polarization as a possible therapeutic target, indicating that it is essential for the development of DCM [5].

Current treatment strategies for DCM primarily focus on pharmacological interventions aimed at glycemic control and lipid-lowering. However, there remains a lack of specific therapeutic agents and standardized management protocols [1]. Traditional Chinese medicine (TCM) is differentiated by its multifocused therapeutic methodology and diverse treatment modalities, along with relatively low incidence of adverse effects [6]. Growing interest in using TCM to treat DCM has been observed in recent years. For instance, Shensong Yangxin capsules have been shown to reduce TGF-*β*1 and p-Smad2/3 expression in diabetic rats' myocardial tissue, thereby improving cardiac function [7]. Qidan Yixin decoction (QDYXD), composed of five herbal ingredients—*Radix Astragali* (Huang Qi), *Radix Angelicae Sinensis* (Dang Gui), *Radix Salviae Liguliobae* (Dan Shen), *Fructus Amomi* (Sha Ren), and *Lignum Santali Albi* (Tan Xiang)—integrates two classical TCM formulas: Dan-Shen decoction and Danggui-Buxue decoction, both traditionally employed in the treatment of cardiovascular diseases [8, 9]. QDYXD has demonstrated extensive clinical use and is recognized for its favorable efficacy and safety profile. Consequently, the exploration of herbal therapies for DCM has emerged as a prominent field of current study interest.

**Figure 1:**
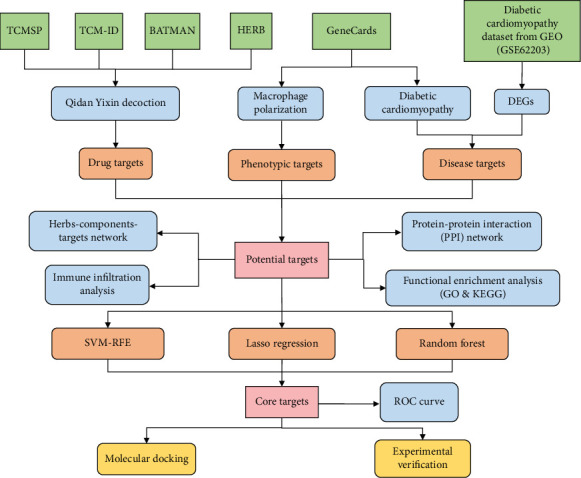
Flow diagram of this study. DEGs, differentially expressed genes; GO, Gene Ontology; KEGG, Kyoto Encyclopedia of Genes and Genomes; SVM-RFE, support vector machine recursive feature elimination; ROC, receiver operating characteristic.

In order to determine QDYXD's active components and valuable targets, this research utilized a network pharmacology technique. Disease-related targets associated with DCM and phenotypic targets related to macrophage polarization were obtained from publicly available databases using bioinformatics techniques. Core targets implicated in QDYXD's treatment of DCM via modulation of macrophage polarization were identified through multiple machine learning algorithms and subsequently validated. In Figure 1, the study's methodology is schematically summarized.

## 2. Materials and Methods

### 2.1. Assessment of QDYXD's Active Ingredients and Objectives

QDYXD consists of five herbal components: *Lignum Santali Albi* (Tan Xiang), *Radix Astragali* (Huang Qi), *Fructus Amomi* (Sha Ren), *Radix Salviae Liguliobae* (Dan Shen), and *Radix Angelicae Sinensis* (Dang Gui). To identify the active constituents, four relevant databases were consulted: Traditional Chinese Medicine Systems Pharmacology (TCMSP) [10], Traditional Chinese Medicine Information Database (TCM-ID) [11], HERB [12], and BATMAN-TCM [13]. The five herbs in QDYXD were identified based on their pharmacological objectives along with active components using oral bioavailability (OB) ≥ 30% and drug-likeness (DL) ≥ 0.18. All identified ingredients along with their associated targets were aggregated and standardized using Microsoft Excel 2024. Following the removal of duplicate entries, a final list of eligible active ingredients and therapeutic targets was compiled.

### 2.2. Screening of Targets Related to DCM and Macrophage Polarization

To identify targets associated with DCM and macrophage polarization, the GeneCards [14] database was queried using the keywords “diabetic cardiomyopathy” and “macrophage polarization,” respectively. From the GEO database, pertinent DCM datasets were also obtained. In R, differential expression analysis was performed utilizing the limma package, with screening norms chosen at |logFC| > 0.585 and *p* < 0.05. To visualize the genes that were differently expressed (DEGs), heatmaps and volcano plots were established. The targets retrieved from the GeneCards database have been merged with the DEGs identified in the GEO datasets. After taking out duplicate entries, a consolidated list of disease-relevant targets for DCM was compiled.

### 2.3. Construct a Network of Herbs–Components–Targets

The convergence of targets linked to disease, pharmaceutical targets, and macrophage polarization has been identified to determine the convergence of key mechanisms. Targets that overlapped were selected as possible QDYXD therapeutic targets for DCM treatment via modulation of macrophage polarization. The results were visualized using a Venn diagram. By combining the active components connected to the discovered potential target, a network of interactions between herbs, components, and targets was created in Cytoscape 3.9.1 [15]. To facilitate the identification of key bioactive compounds, the topological characteristics of target nodes were analyzed using the degree function in the CytoNCA plugin.

### 2.4. Potential Targets' Enrichment Analysis

Enrichment studies of potential targets were carried out utilizing the Kyoto Encyclopedia of Genes and Genomes (KEGG) and Gene Ontology (GO) in R employing the clusterProfiler package. The ggplot2 tool was accustomed to create bubble plots that depicted the eight terms that had the lowest *p* values in the biological process (BP), cellular component (CC), and molecular function (MF) categories. To further observe into the possible therapeutic mechanisms, the 10 most important KEGG pathways featuring the most significant *p* values were selected, and a string diagram has been created to show these pathways.

### 2.5. Development of a Protein–Protein Interaction (PPI) Network

Following their upload to the STRING database [16], the potential targets, since the reference species for building the PPI network, “*Homo sapiens*” was chosen. The obtained data was then imported into the Cytoscape program in order to identify important potential targets within the network. The topological features of the target nodes—such as betweenness, closeness, and degree—were analyzed using the CytoNCA plugin to highlight critical nodes and visualize the network's structural properties.

### 2.6. Immune Infiltration Analysis of Potential Targets

The CIBERSORT algorithm was utilized toward assessing immune cells in the GSE62203 dataset in R software. Box plots were generated for each group for viewing purposes. The association among potential targets with 22 immune cells has been examined via Spearman's correlation coefficient. The data was then visualized using the pheatmap software, which also looked at the association between potential targets and macrophages.

### 2.7. Machine Learning and Receiver Operating Characteristic (ROC) Curve

Gene levels of potential targets were extracted from the GSE62203 dataset to identify the core targets. Support vector machine-recursive feature elimination (SVM-RFE), least absolute shrinkage and selection operator (LASSO) regression, and random forest—three machine learning techniques have—been implemented in order to identify features.

SVM-RFE evaluates the importance of each variable through a support vector machine model, iteratively eliminating less significant variables to obtain an optimal feature subset [17]. To determine the ideal variables, the SVM-RFE approach was assessed using the R software's e1071 and caret packages. LASSO regression performs variable selection by adding an L1 regularization term to the method of least squares, shrinking the coefficients of less relevant variables to zero [18]. The glmnet package was utilized in the implementation of LASSO regression, and the ideal lambda value was chosen via tenfold cross-validation. The random forest algorithm enhances model robustness and predictive performance by constructing an ensemble of decision trees trained on bootstrapped subsets of the dataset. The final predictions are made via majority voting or averaging [19]. A random forest model comprising 500 decision trees was developed using the randomForest package, and genes were ranked according to the MeanDecreaseGini metric.

The three machine learning techniques were illustrated utilizing the ggplot2 program. All three approaches identified genes were regarded as core targets of QDYXD in DCM treatment through macrophage polarization. The pROC package in R was utilized for charting ROC curves in order to assess these core targets' diagnostic efficacy.

### 2.8. Molecular Docking

We identified the primary active constituents of QDYXD for the therapeutic use of DCM by developing a herbs–components–targets network. We acquired the 3-D structures of the primary active constituents via PubChem database [20]. We looked through and obtained the core target protein stereostructures from RCSB database [21]. Protein structures were preprocessed using PyMOL software. The spatial differences in position and conformation between the ligand and receptor molecules were evaluated using root mean square deviation (RMSD). Docking candidates with RMSD values less than 2.0 Å were considered structurally compatible. A suitable grid box was defined to ensure that the entire active site region was encompassed. Binding affinity between the ligands and receptors was assessed by calculating the binding free energy, with favorable binding activity indicated by values below −5 kcal/mol. The PyMOL program was accustomed to visualize molecular docking results of the main active ingredients and core targets using AutoDock Vina function in AutoDockTools (Version 1.5.7).

### 2.9. Establishment of Animal Models

An animal experiment was carried out to evaluate QDYXD's therapeutic efficacy in DCM and elucidate the specific mechanisms of the core targets. The supplier of 30 male Wistar rats (200 ± 20 g, 8 weeks) was Beijing Vital River Laboratory Animal Technology Co. Ltd. A sample size of 10 rats per group is generally considered sufficient to meet statistical requirements, reduce random errors, and fulfill animal ethical standards. The rats were allocated among three groups at random. After a 1-week acclimation period, to induce the DCM model, rats in the DCM and QDYXD groups were fed a diet rich in fat and received one injection of streptozotocin (STZ) intraperitoneally with 100 mg/kg. Four weeks later, successful modeling was confirmed by a reduction of more than 10% in left ventricular ejection fraction (LVEF), as measured by echocardiography, and blood glucose levels during fasting that are higher than 16.7 mmol/L.

The standard clinical dose of QDYXD for a 70-kg adult is 93 g/day. Based on body surface area conversion, the equivalent dosage for rats was calculated as 93 × 0.018 × 5 = 8.37 g/kg/day. Rats in the DCM and control groups received 2 mL of saline via oral gavage daily, while the QDYXD group received 8.37 g/kg/day of QDYXD via gavage. The intervention period for all groups was 8 weeks. Following the anesthesia and euthanasia of the rats, serum and cardiac tissue samples were collected for testing.

### 2.10. Hematoxylin–Eosin (H&E) Staining and Masson Staining

Hematoxylin, an alkaline dye, gives acidic substances, such as nucleic acids within the nucleus, a blue tint, whereas the acidic dye eosin gives cytoplasmic proteins a red tint [22]. We utilized 4% paraformaldehyde to fix rat heart tissue. After the specimen was dehydrated, paraffin was used to embed it. Sliced sections of 4 microns were attached to a glass slide. Hematoxylin and eosin were used to stain the tissue, which was then sealed and seen under a microscope. It is easier to distinguish between collagen fibers, muscle fibers, and other cellular components when using Masson's staining since muscle fibers look red and collagen fibers seem blue [23]. The embedded tissue was sectioned again, stained, dehydrated, sealed, and then viewed under a microscope.

### 2.11. Echocardiography

After pharmaceutical intervention for 8 weeks, pentobarbital was administered intraperitoneally to sedate the rats and then underwent echocardiography. In the left ventricle's short-axis view, the M-curve was measured around the left papillary muscle level. After measuring the left ventricular shortening fraction (LVFS) and LVEF continuously for more than three cardiac cycles, based on the mean values were determined. The values were then plotted in a bar chart for visual analysis.

### 2.12. Quantitative PCR

We extracted RNA from cardiac tissue in each of the three groups and performed qPCR assays. Reverse transcription reagents (Vazyme, R223-01) were utilized to convert RNA into cDNA. Labeling was conducted utilizing SYBR Green (Vazyme, Q311-02). Ultimately, relative quantification was executed utilizing CT results and a standard curve. After that, 2-*ΔΔ*Ct is applied to transform the differences into comparative expressions. Refer to Table S1 for further details regarding the primer sequences.

### 2.13. Western Blotting

The main targets' protein expression levels were found using western blotting. Heart tissue proteins were isolated using RIPA buffer (Servicebio, G2002). The protein concentration has been determined utilizing a BCA protein assay kit (Solarbio, PC0020). SDS-PAGE electrophoresis (Servicebio, G2037) was used to distinguish the proteins. A PVDF membrane received proteins (BIO-RAD, 1620177). The primary antibody was incubated for the entire night (at 4°C) following a 2-h incubation period with 5% skim milk. The image was captured the next day following a 2-h normal temperature incubation period for the secondary antibody, followed by its production using ECL luminol (Vazyme, E412-02). Among the primary antibodies employed in this investigation are PGK1 Rabbit PolyAb (1:5000, Proteintech, 17811-1-AP), HIF1a Polyclonal antibody (1:2000, Proteintech, 20960-1-AP), and LDHA-Specific Rabbit PolyAb (1:2000, Proteintech, 19987-1-AP).

### 2.14. Statistical Analysis

The mean ± standard deviation was accustomed to compiling all of the collected data. For regularly distributed data, a one-way ANOVA was employed, and for nonnormally distributed data, nonparametric tests were employed. SPSS software (Version 27.0) was used to analyze the data, and *p* < 0.05 was deemed statistically significant. The visualization was done using GraphPad Prism (Version 10).

## 3. Results

### 3.1. The Active Components and Targets of QDYXD

By integrating data from four databases—TCMSP, TCMID, HERB, and BATMAN-TCM—a total of 17 active components for Huang Qi, 28 for Dan Shen, 19 for Dang Gui, 21 for Sha Ren, and 11 for Tan Xiang were identified. After removing duplicate compounds, 77 unique active components related to QDYXD were obtained. Similarly, 406 targets were associated with Huang Qi, 428 with Dan Shen, 336 with Dang Gui, 451 with Sha Ren, and 286 with Tan Xiang. Following the removal of duplicate targets, 924 unique herb-related targets were retained for QDYXD. Table S2 provides comprehensive information on each herb's active ingredients and targets.

### 3.2. Target Related to DCM and Macrophage Polarization

**Figure 2:**
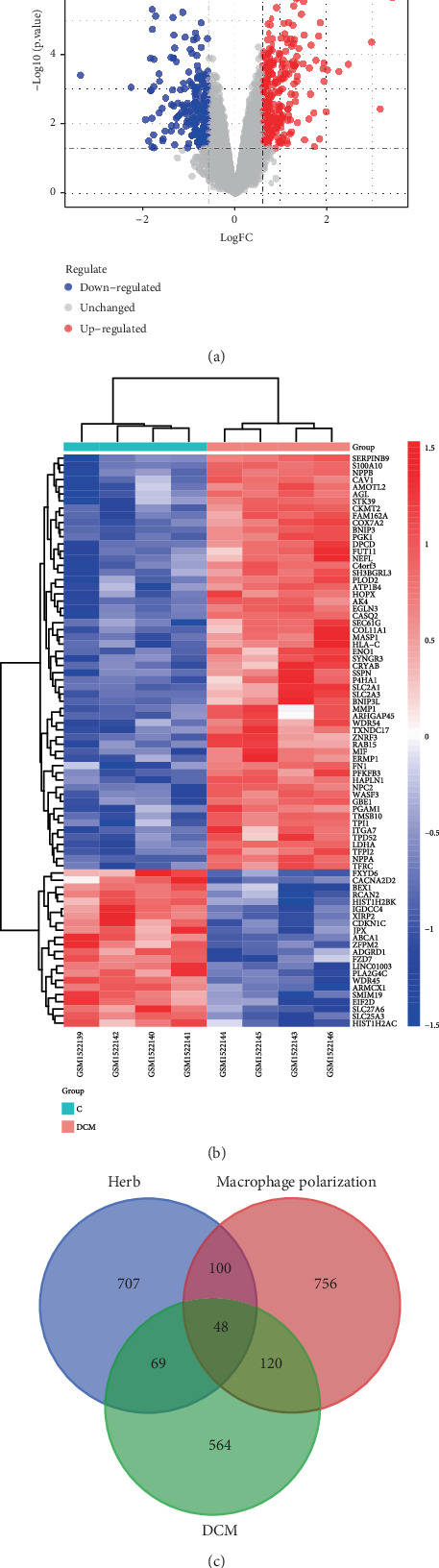
Differential expression analysis of the DCM dataset. (a) Volcano plot of DEGs in GSE62203. (b) Heatmap of DEGs in GSE62203. (c) Venn diagram of the intersection of DCM, macrophage polarization, and drug targets for QDYXD. QDYXD, Qidan Yixin decoction.

A total of 300 disease-related targets associated with DCM and 1024 phenotype-related targets were obtained via the GeneCards database and linked to macrophage polarization. After evaluating sample characteristics, data completeness, and analytical feasibility across available DCM datasets in the GEO database, the GSE62203 dataset was chosen for further analysis. Utilizing the R software for differential expression analysis and applying screening criteria of *p* < 0.05 and |logFC| > 0.585, 284 upregulated and 231 downregulated genes have been identified. These DEGs have been depicted using a heatmap and volcano plot (Figure 2a,b). Following the removal of duplicate data, 801 disease targets were found by combining the DCM-related genes from the GeneCards database and DEGs.

### 3.3. Herbs–Components–Targets Network

**Figure 3:**
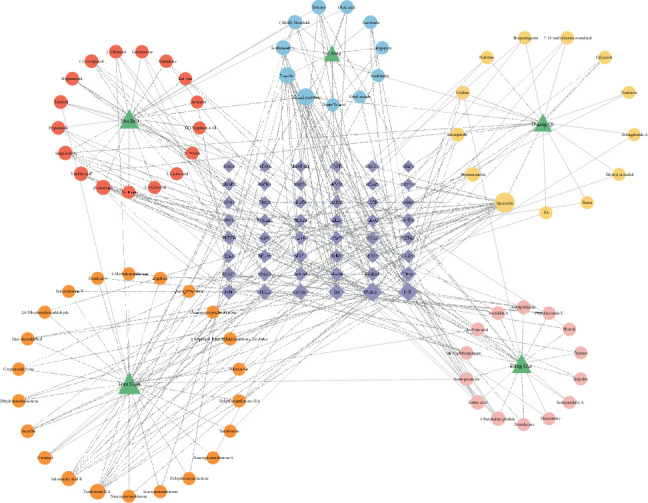
The herbs–components–targets network of 48 potential targets.

By intersecting 924 herb-related targets, 801 disease-related targets, and 1024 phenotype-related targets, 48 potential targets associated with QDYXD for the treatment of DCM via macrophage polarization were identified (Figure 2c). The active components of herbs linked to these 48 potential targets were looked up, and the herbs–components–targets network was constructed and delivered via Cytoscape (Figure 3). The resulting network contained 130 nodes and 304 edges. Among the five herbs, Dan Shen exhibited the highest degree centrality, demonstrating its important position within the network. Four active components—quercetin, methyl palmitate, luteolin, and tanshinone IIA—also showed high degree scores and were identified as key active constituents of QDYXD.

### 3.4. GO and KEGG Enrichment Analysis

**Figure 4:**
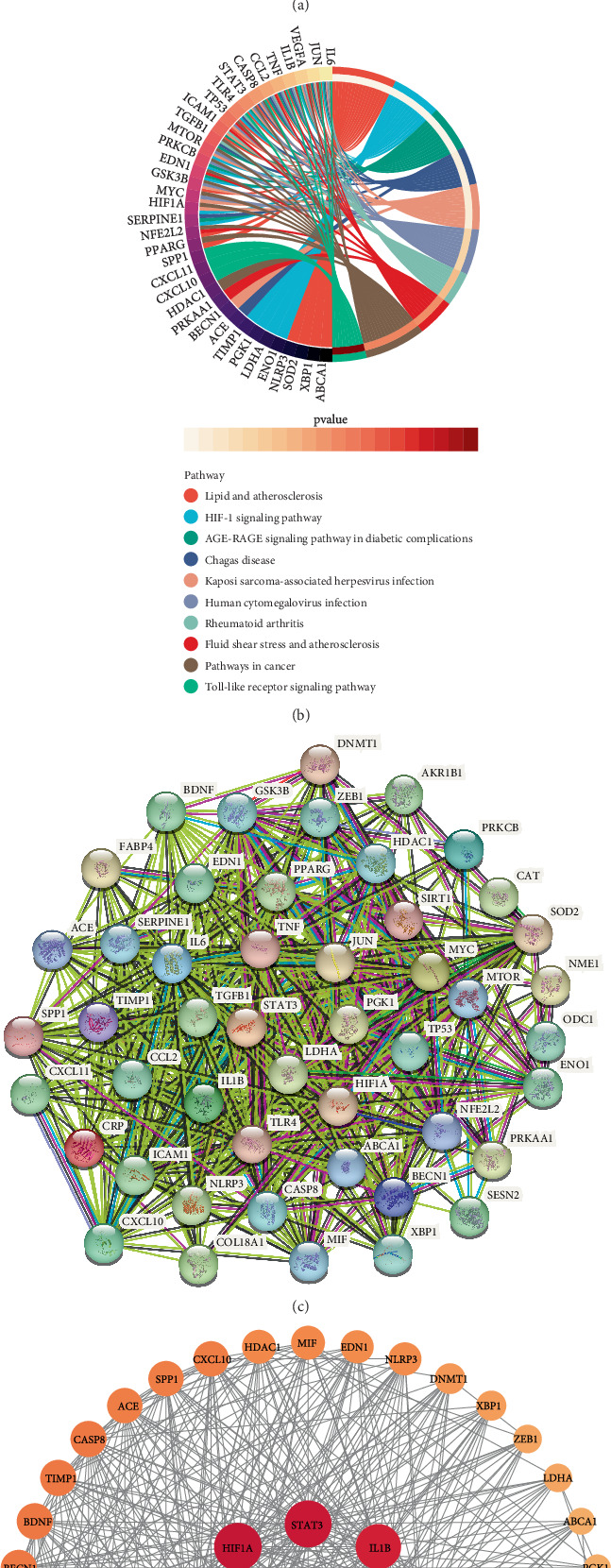
Functional analysis and PPI network of potential targets. (a) Bubble diagram of GO analysis. (b) Chord diagram of KEGG analysis. (c) PPI network of potential targets. (d) Network diagram of potential targets with degree centrality.

Utilizing a bubble chart (Figure 4a), the top eight results from the three approaches were shown—BP, CC, and MF—that were most significantly enriched. Among them, BP was mainly enriched in positive regulation of gene expression, negative regulation of apoptotic process, and cellular response to hypoxia. The extracellular space, cytoplasm, and extracellular region were where CC was most abundant. MF was primarily enriched in protease binding, cytokine activity, and identical protein binding. Signaling pathway mechanisms associated with 48 potential targets were investigated using KEGG analysis. The findings centered on the AGE-RAGE signaling route, HIF-1 signaling pathway, and lipid and atherosclerosis in diabetes complications. To visualize the top 10 routes in the KEGG results, we created a string plot (Figure 4b).

### 3.5. Construction of PPI Network

A network graph with 541 edges and 48 nodes was created after selecting the interaction scores greater than 0.4 and *p* values less than 0.05 (Figure 4c). After importing the PPI network into Cytoscape, degree, betweenness, and closeness, among other network topology metrics, were calculated. Based on their degree scores, the nodes of the network were positioned and shown; the more significant a node was in DCM, the darker its color (Figure 4d). Genes TP53, TNF, IL6, HIF1A, STAT3, IL1B, TGFB1, JUN, PPARG, and SIRT1 were among the top 10.

### 3.6. Immune Infiltration Analysis

**Figure 5:**
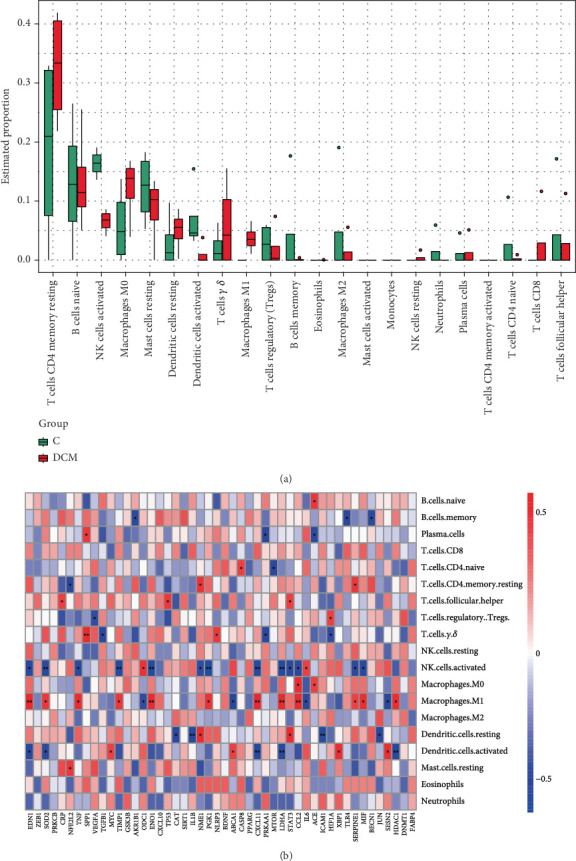
Immune infiltration analysis based on the GSE62203 dataset. (a) Box diagram of immune infiltrating cells in control and DCM. (b) Heatmap of the correlation between potential targets and immune cells. ⁣^∗^*p* < 0.05.

The outcomes are displayed in a box graph (Figure 5a), which revealed that T cells, macrophages, NK cells, B cells, and mast cells were the main immune cells implicated in DCM. M1 macrophages and activated NK cells exhibited statistically notable variations among the control and DCM groups (*p* < 0.05), with M1 macrophages being more abundant in the DCM group. To further explore immune regulation, a heatmap (Figure 5b) was generated to examine the correlations between the 22 immune cell types and the identified potential targets. Twelve targets—EDN1, SOD2, TNF, TIMP1, ENO1, PGK1, CXCL11, LDHA, CCL2, SERPINE1, MIF, and HDAC1—were found to be significantly positively correlated (*p* < 0.05) with M1 macrophage levels.

### 3.7. Machine Learning and ROC Curve

**Figure 6:**
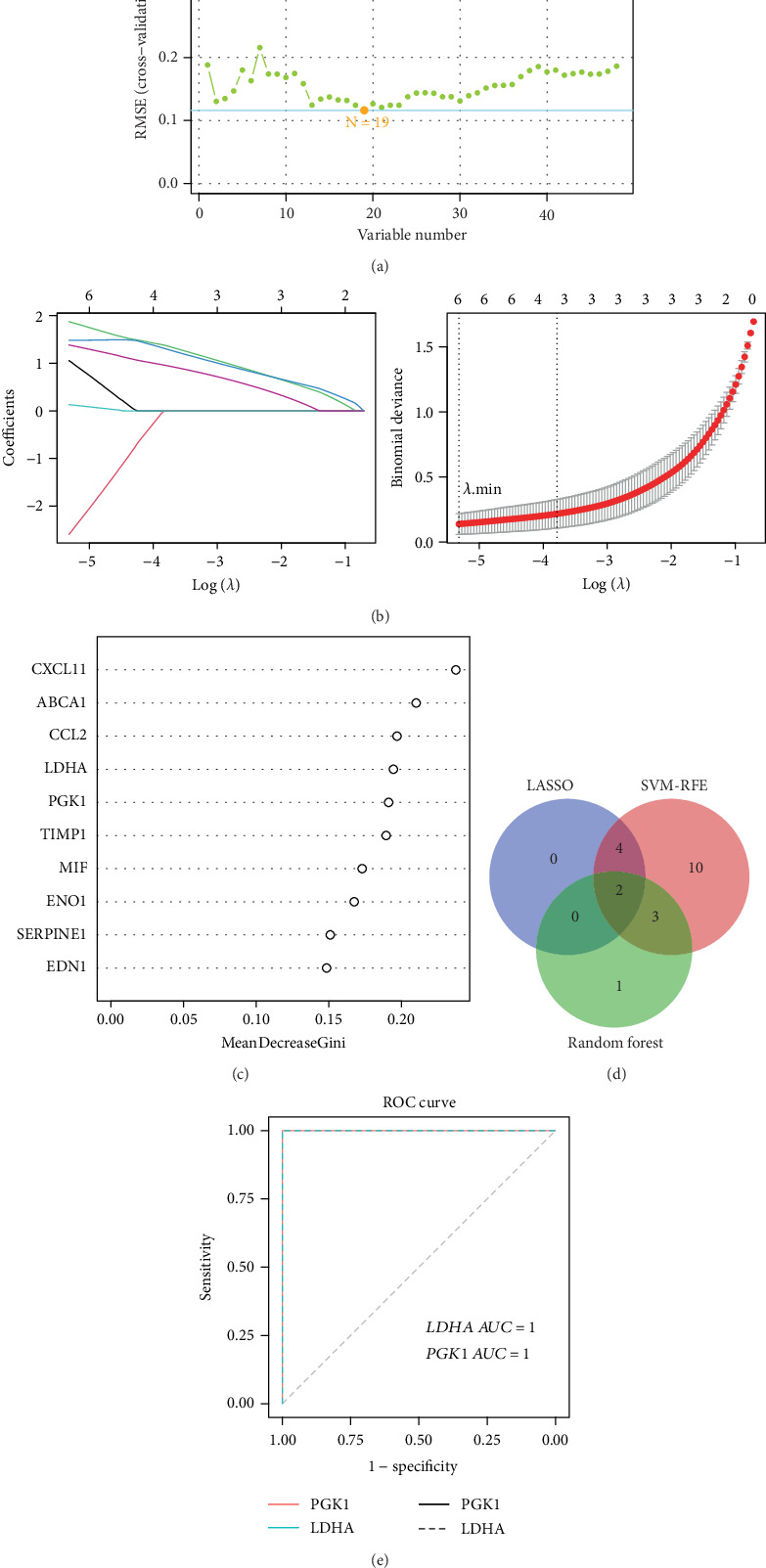
Machine learning to screen for core targets. (a) The relationship between the number of variables and the accuracy of the cross-validation. (b) LASSO logistic regression coefficient path diagram (left). The most suitable lambda value in the LASSO model (right). (c) The top 10 targets selected by random forest. (d) Venn diagram of three machine learning methods for screening core targets. (e) ROC curves for the core targets.

Based on the SVM-RFE results, 19 candidate targets were identified at the point of minimum root mean squared error (RMSE) (Figure 6a), including LDHA, PGK1, MIF, EDN1, CCL2, ABCA1, ENO1, SERPINE1, ODC1, CASP8, MTOR, GSK3B, CXCL11, TLR4, NME1, SIRT1, CRP, HDAC1, and ZEB1. LASSO regression, using tenfold cross-validation and the optimal lambda value, yielded six selected genes: EDN1, ZEB1, PGK1, LDHA, SERPINE1, and MIF (Figure 6b). In the random forest model, genes were ranked by the MeanDecreaseGini coefficient, and the top 10 were visualized (Figure 6c). Six genes with MeanDecreaseGini values greater than 0.18—CXCL11, ABCA1, CCL2, LDHA, PGK1, and TIMP1—were selected. PGK1 and LDHA were used as the core targets after the intersection. The outcomes were displayed as a Venn diagram (Figure 6d). The ROC curves were employed according to their expression levels in the GSE62203 dataset. Excellent diagnostic performance was demonstrated by both PGK1 and LDHA, which both had an AUC value of 1.0 (Figure 6e).

### 3.8. Molecular Docking

**Figure 7:**
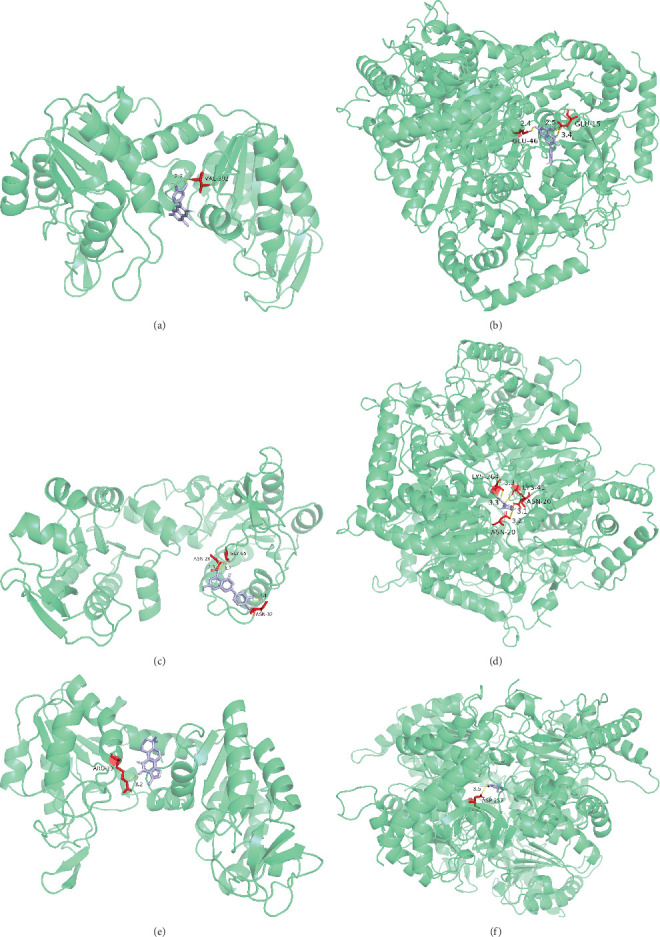
Molecular docking results of the main active components of QDYXD and the core targets. (a) PGK1-quercetin; (b) LDHA-quercetin; (c) PGK1-luteolin; (d) LDHA-luteolin; (e) PGK1-tanshinone IIA; (f) LDHA-tanshinone IIA.

Based on the herbs–components–targets network, quercetin, methyl palmitate, luteolin, and tanshinone IIA were identified as the primary active components of QDYXD. The RCSB database provided the protein structures of PGK1 and LDHA, while the PubChem database provided the three-dimensional structures of the primary active ingredients. Molecular docking was performed using AutoDockTools after preprocessing the protein structures with PyMOL software. The docking results indicated stable binding affinities between PGK1 and quercetin (−7.1 kcal/mol), luteolin (−7.1 kcal/mol), and tanshinone IIA (−7.6 kcal/mol). Similarly, LDHA showed strong binding with luteolin (−8.0 kcal/mol), quercetin (−7.7 kcal/mol), and tanshinone IIA (−8.2 kcal/mol). All of these binding strengths were lower than −5 kcal/mol, suggesting favorable interactions. In contrast, methyl palmitate exhibited poor docking performance with both PGK1 and LDHA. The docking conformations were visualized using PyMOL (Figure 7).

### 3.9. Animal Experiment

**Figure 8:**
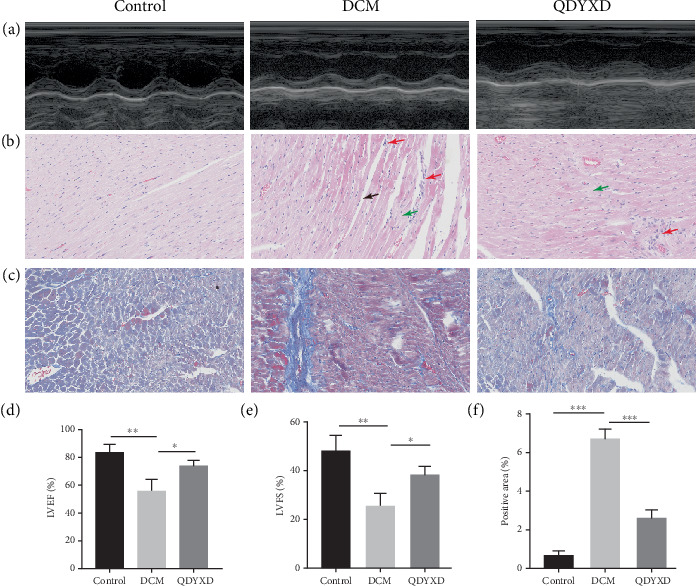
The results of animal experiments with QDYXD to improve DCM myocardial injury, fibrosis, and heart function. (a) Representative echocardiogram images. (b) H&E staining representative images. (c) Masson staining representative images. (d) Results of statistical analysis of LVEF. (e) Results of statistical analysis of LVFS. (f) Results of statistical analysis of collagen fiber area by Masson staining. LVEF, left ventricular ejection fraction; LVFS, left ventricular short axis shrinkage. ⁣^∗^*p* < 0.05, ⁣^∗∗^*p* < 0.01, and ⁣^∗∗∗^*p* < 0.001.

Cardiac function in rats was evaluated using echocardiography, with representative images as seen in Figure 8a. The DCM group exhibited substantially decreased LVEF and LVFS (*p* < 0.01). However, treatment with QDYXD significantly improved both LVEF and LVFS levels in contrast to the DCM group (*p* < 0.05) (Figure 8d,e). Histological examination utilizing H&E staining disclosed evenly stained myocardial fibers, clear cell boundaries, and consistent orientation in the control group. Myocardial cell striations were distinct, and no indications of inflammation or necrosis were observed. In the contrary, the DCM group disclosed myocardial fiber loss (black arrows), watery degeneration in some cardiomyocytes (green arrows) with swollen cells and pale cytoplasm, and focal inflammatory cell infiltration (red arrows). Myocardial cells had eosinophilic cytoplasm and irregular, shrunken nuclei. In the QDYXD-treated group, most myocardial fibers were uniformly stained and regularly arranged with clear cell borders. Mild inflammatory cell infiltration (red arrows) and occasional cell swelling (green arrows) were observed, but the interstitial tissue appeared normal. Myocardial cells demonstrated distinct transverse striations and alternating light and dark bands (Figure 8b). Masson staining showed limited fibrosis in the control group (Figure 8c). The QDYXD group had a considerably smaller area of cardiac fibrosis (*p* < 0.001) (Figure 8f). The aforementioned findings demonstrate that with QDYXD administration, the rat's ventricular systolic performance considerably improved, and myocardial damage and fibrosis decreased.

**Figure 9:**
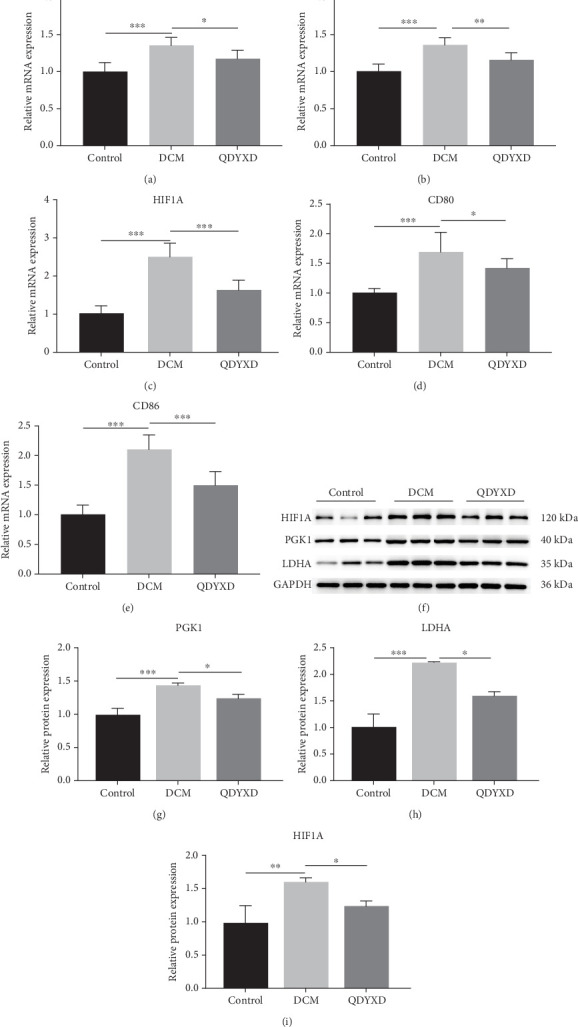
Results of qPCR and western blotting on rat models. The mRNA relative expression levels of (a) PGK1, (b) LDHA, and (c) HIF1A. The mRNA relative expression levels of macrophage polarization markers (d) CD80 and (e) CD86. (f) Representative strips of western blotting results. The protein relative expression levels of (g) PGK1, (h) LDHA, and (i) HIF1A. ⁣^∗^*p* < 0.05, ⁣^∗∗^*p* < 0.01, and ⁣^∗∗∗^*p* < 0.001.

According to earlier research, the HIF-1 signaling pathway contributes substantially to DCM management by macrophage polarization with QDYXD. Specifically, the core targets PGK1, LDHA, and HIF1A have been shown to positively correlate with M1 macrophage polarization. The results of the qPCR showed that the relative mRNA expression levels of PGK1, LDHA, and HIF1A in the DCM group were substantially higher (*p* < 0.001). Treatment with QDYXD significantly reduced the expression levels of PGK1 (*p* < 0.05), HIF1A (*p* < 0.001), and LDHA (*p* < 0.01) in contrast to the DCM group (Figures 9a, 9b, and 9c). The DCM group had significantly greater comparative mRNA expression values of CD80 and CD86, markers of M1 macrophage polarization (*p* < 0.001). Following QDYXD therapy, their expression levels were notably reduced (*p* < 0.05 for CD80; *p* < 0.001 for CD86) (Figure 9d,e). Western blot analysis further confirmed these findings. Representative protein strips can be observed in Figure 9f. Relative protein expression levels of PGK1 (*p* < 0.001), LDHA (*p* < 0.001), and HIF1A (*p* < 0.01) were notably upregulated in the DCM group, while QDYXD treatment significantly suppressed their expression (*p* < 0.05 for all comparisons) (Figures 9g, 9h, and 9i). Collectively, these results indicate that QDYXD reduces M1 macrophage polarization and inhibits the HIF-1 signaling pathway by downregulating PGK1, LDHA, and HIF1A expression, thereby improving cardiac function in DCM.

## 4. Discussion

DCM is clinically classified into two primary stages. During the first phase, diastolic dysfunction and left ventricular hypertrophy are the primary characteristics. The main characteristics of the second phase are cardiac fibrosis and dysfunction of the systolic valve [1]. According to current understanding, the pathophysiology of DCM is mostly associated with anomalies in endothelial dysfunction, inflammation, oxidative stress, late glycosylation end-product production, apoptosis, and glucose and lipid metabolism [24]. Myocardial inflammation in DCM is closely linked to macrophage polarization. Circulating monocytes are recruited to the injured myocardium, where they differentiate into M1 macrophages and exacerbate the inflammatory response [25]. Some studies have found that in T2DM mice with decreased cardiac function, an imbalance of macrophage polarization occurs, and M1 macrophage infiltration predominates in cardiac tissue, suggesting that macrophage polarization-mediated inflammatory responses have an essential role in DCM [5]. Further investigation into the role of macrophage polarization in DCM pathophysiology may offer novel therapeutic strategies. The application of TCM as an alternative to DCM has gained popularity in recent years. QDYXD, a TCM formulation widely used in clinical practice for cardiovascular diseases, warrants in-depth study to elucidate its mechanisms in DCM therapy.

In this study, utilizing network pharmacology and bioinformatics techniques, we first identified the primary active ingredients and pharmacological targets of QDYXD in four databases: TCMSP, TCMID, HERB, and BATMAN-TCM. Macrophage polarization's phenotypic targets have been identified in the GeneCards database. DEGs associated with DCM were identified through differential expression analysis of the GSE62203 dataset and subsequently integrated with DCM-related targets from GeneCards to generate a comprehensive set of disease-related genes. The potential targets of QDYXD for treating DCM via macrophage polarization were then identified by intersecting the herb-related targets, disease-related genes, and macrophage polarization targets.

By constructing a herbs–components–targets network, four principal active components of QDYXD were identified: quercetin, methyl palmitate, luteolin, and tanshinone IIA. GO and KEGG enrichment analyses disclosed that the potential targets were primarily involved in the lipid and atherosclerosis pathway, the HIF-1 signaling pathway, and the AGE-RAGE signaling pathway associated with diabetic complications. The PPI network analysis ranked the potential targets based on topological parameters to indicate their relative importance. According to the examination of immune infiltration, the DCM group had higher levels of M1 macrophage expression than the control group. In addition, 12 genes showed a favorable correlation with M1 macrophages, including PGK1 and LDHA. Two core targets, PGK1 and LDHA, were eliminated using the machine learning techniques of SVM-FRE, LASSO, and random forest. Molecular docking results indicated that quercetin, luteolin, and tanshinone IIA exhibited stable binding affinities with both PGK1 and LDHA, suggesting these components might be quite significant in treating DCM by modulating macrophage polarization.

To validate the therapeutic efficacy of QDYXD in DCM and its effect on core targets and the HIF-1 signaling pathway, in vivo experiments were conducted. The experiment results demonstrated that QDYXD significantly enhanced ventricular contractility in DCM rats. Structurally, QDYXD markedly reduced cardiomyocyte death and myocardial fibrosis. Mechanistically, QDYXD downregulated the expression of PGK1, LDHA, and HIF1A, thereby preventing the HIF-1 signaling pathway from being activated in DCM rats. Furthermore, QDYXD decreased M1 macrophage markers CD80 and CD86 expression. According to this study, QDYXD can prevent the overexpression of PGK1, LDHA, and HIF1A, prevent M1 macrophage polarization, reduce myocardial damage and fibrosis in DCM, and preserve cardiac function.

There are several studies that have looked into how the three main active components in QDYXD work to combat DCM by polarizing macrophages. Quercetin has demonstrated antioxidative, anti-inflammatory, and regulatory effects on cell proliferation and apoptosis [26]. Wang et al. reported that quercetin increases superoxide dismutase (SOD) levels, preserves mitochondrial function, reduces H9C2 cardiomyocyte apoptosis and inflammation, and activates the Nrf2/HO-1 signaling pathway [27]. Similarly, Chen et al. found that quercetin enhances cardiomyocyte autophagy, inhibits apoptosis, and alleviates myocardial injury [28]. Luteolin, a natural flavonoid, has been shown to protect the myocardium of diabetic rats from ischemia–reperfusion injury by attenuating oxidative stress [29]. According to Xiao et al., luteolin administration resulted in a considerable improvement in cardiac function in diabetic mice, autophagy, myocardial fibrosis, and the downregulation of both miR-221 and JNK/c-Jun in hearts suffering from diabetes [30]. The primary active ingredient in DAN SHEN, a traditional Chinese medication, is tanshinone IIA. Because of its strong pharmacological action in both preventing and treating cardiovascular disease, it has been extensively researched. It also has anticancer, antioxidant, anticoagulant, and hypoglycemic effects [31]. Notably, tanshinone IIA has been reported to alleviate DCM by inhibiting endoplasmic reticulum stress, stimulating SIRT1 expression, and improving myocardial structure and function [32]. In conclusion, these three active components exhibit diverse mechanisms in cardiovascular protection. Further study will focus on the specific mechanism of treating DCM by macrophage polarization through a single active component.

This study demonstrates that QDYXD can suppress M1 macrophage polarization and inhibit the HIF-1 signaling pathway's activation. Several studies have explored the interplay between macrophage polarization and HIF-1 signaling. Transcription factors like hypoxia-inducible factors (HIFs) can activate macrophages, which are crucial for systemic inflammatory and immunological responses. In particular, HIF1A has been shown to promote M1 macrophage polarization by regulating glucose metabolism and thereby enhancing the inflammatory response [33]. Zeng et al. reported that phloretin inhibits HIF1A-mediated glycolysis, restores the M1/M2 macrophage polarization balance, and attenuates the proinflammatory effects of M1 macrophages [34]. PGK1, a key glycolytic enzyme and major effector in the HIF-1 signaling pathway, has also been implicated in macrophage polarization. According to certain research, PGK1 expression inhibition can reduce inflammation by preventing M1 macrophage polarization and macrophage pyrolysis [35]. In the hyperglycemic environment characteristic of DCM, PGK1 expression is upregulated, potentially increasing glycolytic flux and providing metabolic support for M1 macrophage polarization. PGK1 facilitates proinflammatory cytokine expression associated with M1 polarization by regulating glycolysis and mitochondrial function, thereby activating inflammatory signaling pathways. LDHA is a metabolic molecule associated with the glycolytic system that can regulate cell division. According to certain research, when macrophages lack LDHA, inflammation is considerably decreased and anti-inflammatory pathways are activated [36]. Diao et al. demonstrated that LDHA expression, M1 macrophage polarization, and inflammatory cytokine production may all be decreased by inhibiting HIF1A [37]. In DCM, high glucose levels induce elevated LDHA expression, resulting in increased lactate production. Lactate, in turn, promotes the expression of M1 macrophage–associated proinflammatory genes by activating the HIF-1*α* signaling pathway. Thus, LDHA-mediated lactate production may reprogram macrophage metabolism and immune function, driving polarization in the direction of the M1 phenotype. The present findings reinforce an association between the HIF-1 signaling pathway and macrophage polarization in DCM. Nevertheless, further research is warranted to fully elucidate the mechanistic relationships among LDHA, PGK1, and M1 macrophage polarization.

Although this study was designed and implemented rigorously and has yielded valuable findings, several limitations remain. First, while TCM demonstrates promising clinical efficacy, its precise molecular mechanisms remain largely unclear and warrant further investigation. Second, the number of disease-related targets for DCM available in current databases is relatively limited, which may impact the comprehensiveness and accuracy of the results. Although the roles of PGK1 and LDHA in macrophage polarization during DCM are well supported, this study lacks experimental validation through siRNA knockdown or inhibitor-based assays. Moreover, the connection between DCM and macrophage polarization is poorly understood, and our findings represent only a preliminary exploration and validation. Positive controls, like metformin or SGLT2 inhibitors, should be used in future research to compare the therapeutic effectiveness of QDYXD. Additionally, further mechanistic studies are required in order to explain the roles of QDYXD's key active components in DCM therapy.

## 5. Conclusion

This study preliminarily elucidated the mechanism by which QDYXD treats DCM through modulation of macrophage polarization. The active components quercetin, luteolin, and tanshinone IIA were identified as potential key therapeutic agents. The polarization of macrophages is significantly influenced by the HIF-1 signaling pathway. QDYXD may exert its therapeutic effects by targeting PGK1 and LDHA, thereby inhibiting M1 macrophage polarization. Furthermore, QDYXD downregulates the gene expression pertaining to the HIF-1 signaling pathway (PGK1, LDHA, and HIF1A), suppresses M1 macrophage polarization, and improves cardiac function in DCM model rats.

## Data Availability

The dataset (GSE62203) analyzed in this paper was downloaded from the NCBI-GEO database (https://www.ncbi.nlm.nih.gov/GEO/). Other public data were from the following databases: TCMSP (https://old.tcmsp-e.com/), TCM-ID (https://www.bidd.group/TCMID/), HERB (http://herb.ac.cn/), BATMAN-TCM (http://bionet.ncpsb.org.cn/batman-tcm/), GeneCards (https://www.genecards.org/), String (https://cn.string-db.org/), PubChem (https://pubchem.ncbi.nlm.nih.gov/), and RCSB (https://www.rcsb.org/).

## Supporting Information

Supporting Information Additional supporting information can be found online in the Supporting Information section. Figure S1. Western blotting original strips. (A) GAPDH; (B) PGK1; (C) LDHA; (D) HIF1A. Table S1. Gene-specific primers utilized in qPCR. Table S2. Active components and targets of herbs.

## References

[1] Tan Y., Zhang Z., Zheng C., Wintergerst K. A., Keller B. B., Cai L. (2020). Mechanisms of Diabetic Cardiomyopathy and Potential Therapeutic Strategies: Preclinical and Clinical Evidence. *Nature Reviews Cardiology*.

[2] Januzzi J. L., del Prato S., Rosenstock J. (2024). Characterizing Diabetic Cardiomyopathy: Baseline Results From the ARISE-HF Trial. *Cardiovascular Diabetology*.

[3] Bouthoorn S., Valstar G. B., Gohar A. (2018). The Prevalence of Left Ventricular Diastolic Dysfunction and Heart Failure With Preserved Ejection Fraction in Men and Women With Type 2 Diabetes: A Systematic Review and Meta-Analysis. *Diabetes and Vascular Disease Research*.

[4] Prame Kumar K., Nicholls A. J., Wong C. H. Y. (2018). Partners in Crime: Neutrophils and Monocytes/Macrophages in Inflammation and Disease. *Cell and Tissue Research*.

[5] Zhang S., Zhu X., Chen Y., Wen Z., Shi P., Ni Q. (2024). The Role and Therapeutic Potential of Macrophages in the Pathogenesis of Diabetic Cardiomyopathy. *Frontiers in Immunology*.

[6] Li W., Liu X., Liu Z. (2024). The Signaling Pathways of Selected Traditional Chinese Medicine Prescriptions and Their Metabolites in the Treatment of Diabetic Cardiomyopathy: A Review. *Frontiers in Pharmacology*.

[7] Shen N., Li X., Zhou T. (2014). Shensong Yangxin Capsule Prevents Diabetic Myocardial Fibrosis by Inhibiting TGF-*β*1/Smad Signaling. *Journal of Ethnopharmacology*.

[8] Li D., Si J., Guo Y. (2023). Danggui-Buxue Decoction Alleviated Vascular Senescence in Mice Exposed to Chronic Intermittent Hypoxia Through Activating the Nrf2/HO-1 Pathway. *Pharmaceutical Biology*.

[9] Gong D., Yuan T., Wang R. (2023). Network Pharmacology Approach and Experimental Verification of Dan-Shen Decoction in the Treatment of Ischemic Heart Disease. *Pharmaceutical Biology*.

[10] Ru J., Li P., Wang J. (2014). TCMSP: A Database of Systems Pharmacology for Drug Discovery From Herbal Medicines. *Journal of Cheminformatics*.

[11] Chen X., Zhou H., Liu Y. B. (2006). Database of Traditional Chinese Medicine and Its Application to Studies of Mechanism and to Prescription Validation. *British Journal of Pharmacology*.

[12] Fang S., Dong L., Liu L. (2021). HERB: A High-Throughput Experiment- and Reference-Guided Database of Traditional Chinese Medicine. *Nucleic Acids Research*.

[13] Kong X., Liu C., Zhang Z. (2024). BATMAN-TCM 2.0: An Enhanced Integrative Database for Known and Predicted Interactions Between Traditional Chinese Medicine Ingredients and Target Proteins. *Nucleic Acids Research*.

[14] Stelzer G., Rosen N., Plaschkes I. (2016). The GeneCards Suite: From Gene Data Mining to Disease Genome Sequence Analyses. *Current Protocols in Bioinformatics*.

[15] Otasek D., Morris J. H., Bouças J., Pico A. R., Demchak B. (2019). Cytoscape Automation: Empowering Workflow-Based Network Analysis. *Genome Biology*.

[16] Szklarczyk D., Kirsch R., Koutrouli M. (2023). The STRING Database in 2023: Protein-Protein Association Networks and Functional Enrichment Analyses for Any Sequenced Genome of Interest. *Nucleic Acids Research*.

[17] Sanz H., Valim C., Vegas E., Oller J. M., Reverter F. (2018). SVM-RFE: Selection and Visualization of the Most Relevant Features Through Non-Linear Kernels. *BMC Bioinformatics*.

[18] Wong A., Kramer S. C., Piccininni M. (2023). Using LASSO Regression to Estimate the Population-Level Impact of Pneumococcal Conjugate Vaccines. *American Journal of Epidemiology*.

[19] Hu J., Szymczak S. (2023). A Review on Longitudinal Data Analysis With Random Forest. *Briefings in Bioinformatics*.

[20] Kim S., Chen J., Cheng T. (2023). PubChem 2023 Update. *Nucleic Acids Research*.

[21] Bittrich S., Segura J., Duarte J. M., Burley S. K., Rose Y. (2024). RCSB Protein Data Bank: Exploring Protein 3D Similarities via Comprehensive Structural Alignments. *Bioinformatics*.

[22] Feldman A. T., Wolfe D. (2014). Tissue Processing and Hematoxylin and Eosin Staining. *Methods in Molecular Biology*.

[23] Van De Vlekkert D., Machado E., d’Azzo A. (2020). Analysis of Generalized Fibrosis in Mouse Tissue Sections With Masson’s Trichrome Staining. *Bio-Protocol*.

[24] Wang M., Li Y., Li S., Lv J. (2022). Endothelial Dysfunction and Diabetic Cardiomyopathy. *Frontiers in Endocrinology*.

[25] Liu G., Yan D., Yang L. (2021). The Effect of miR-471-3p on Macrophage Polarization in the Development of Diabetic Cardiomyopathy. *Life Sciences*.

[26] Xi Y., Shen J., Li X. (2023). Regulatory Effects of Quercetin on Bone Homeostasis: Research Updates and Future Perspectives. *American Journal of Chinese Medicine*.

[27] Wang S. H., Tsai K. L., Chou W. C. (2022). Quercetin Mitigates Cisplatin-Induced Oxidative Damage and Apoptosis in Cardiomyocytes Through Nrf2/HO-1 Signaling Pathway. *American Journal of Chinese Medicine*.

[28] Chen Y. F., Qiu Q., Wang L. (2024). Quercetin Ameliorates Myocardial Injury in Diabetic Rats by Regulating Autophagy and Apoptosis Through AMPK/mTOR Signaling Pathway. *American Journal of Chinese Medicine*.

[29] Zhou X. R., Ru X. C., Xiao C. (2021). Sestrin2 Is Involved in the Nrf2-Regulated Antioxidative Signaling Pathway in Luteolin-Induced Prevention of the Diabetic Rat Heart From Ischemia/Reperfusion Injury. *Food & Function*.

[30] Xiao C., Chen M. Y., Han Y. P., Liu L. J., Yan J. L., Qian L. B. (2023). The Protection of Luteolin Against Diabetic Cardiomyopathy in Rats Is Related to Reversing JNK-Suppressed Autophagy. *Food & Function*.

[31] Ansari M. A., Khan F. B., Safdari H. A. (2021). Prospective Therapeutic Potential of Tanshinone IIA: An Updated Overview. *Pharmacological Research*.

[32] Wu S., Lu D., Gajendran B. (2023). Tanshinone IIA ameliorates Experimental Diabetic Cardiomyopathy by Inhibiting Endoplasmic Reticulum Stress in Cardiomyocytes via SIRT1. *Phytotherapy Research*.

[33] Qiu B., Yuan P., Du X., Jin H., Du J., Huang Y. (2023). Hypoxia Inducible Factor-1*α* Is an Important Regulator of Macrophage Biology. *Heliyon*.

[34] Zeng X., Li T., Yang K. (2024). Natural Compound Phloretin Restores Periodontal Immune Homeostasis via HIF-1*α*-Regulated PI3K/Akt and Glycolysis in Macrophages. *International Immunopharmacology*.

[35] Zhu G., Yu H., Peng T., Yang K., Xu X., Gu W. (2024). Glycolytic Enzyme PGK1 Promotes M1 Macrophage Polarization and Induces Pyroptosis of Acute Lung Injury via Regulation of NLRP3. *Respiratory Research*.

[36] Lu Y., Osis G., Zmijewska A. A. (2025). Macrophage-Specific Lactate Dehydrogenase Expression Modulates Inflammatory Function In Vitro. *Kidney*.

[37] Diao R. X., Lv W. Y., Wang Y. C. (2024). Aquaporin-1 Facilitates Macrophage M1 Polarization by Enhancing Glycolysis Through the Activation of HIF1*α* in Lipopolysaccharide-Induced Acute Kidney Injury. *Inflammation*.

