# Supplementary material for: Mechanistic Insights Into Qidan Yixin Decoction for Diabetic Cardiomyopathy via Macrophage Polarization
Source: J Diabetes Res. 2025 Jul 31;2025:7578626. doi: 10.1155/jdr/7578626 (PMC12331402; doi:10.1155/jdr/7578626)
Supplement: Supporting Information — Additional supporting information can be found online in the Supporting Information section. Figure S1. Western blotting original strips. (A) GAPDH; (B) PGK1; (C) LDHA; (D) HIF1A. Table S1. Gene-specific primers utilized in qPCR. Table S2. Active components and targets of herbs. [file 7578626.f1.docx]

Fig S1. Original strips from western blotting. (A) GAPDH; (B) PGK1; (C) LDHA; (D) HIF1A.


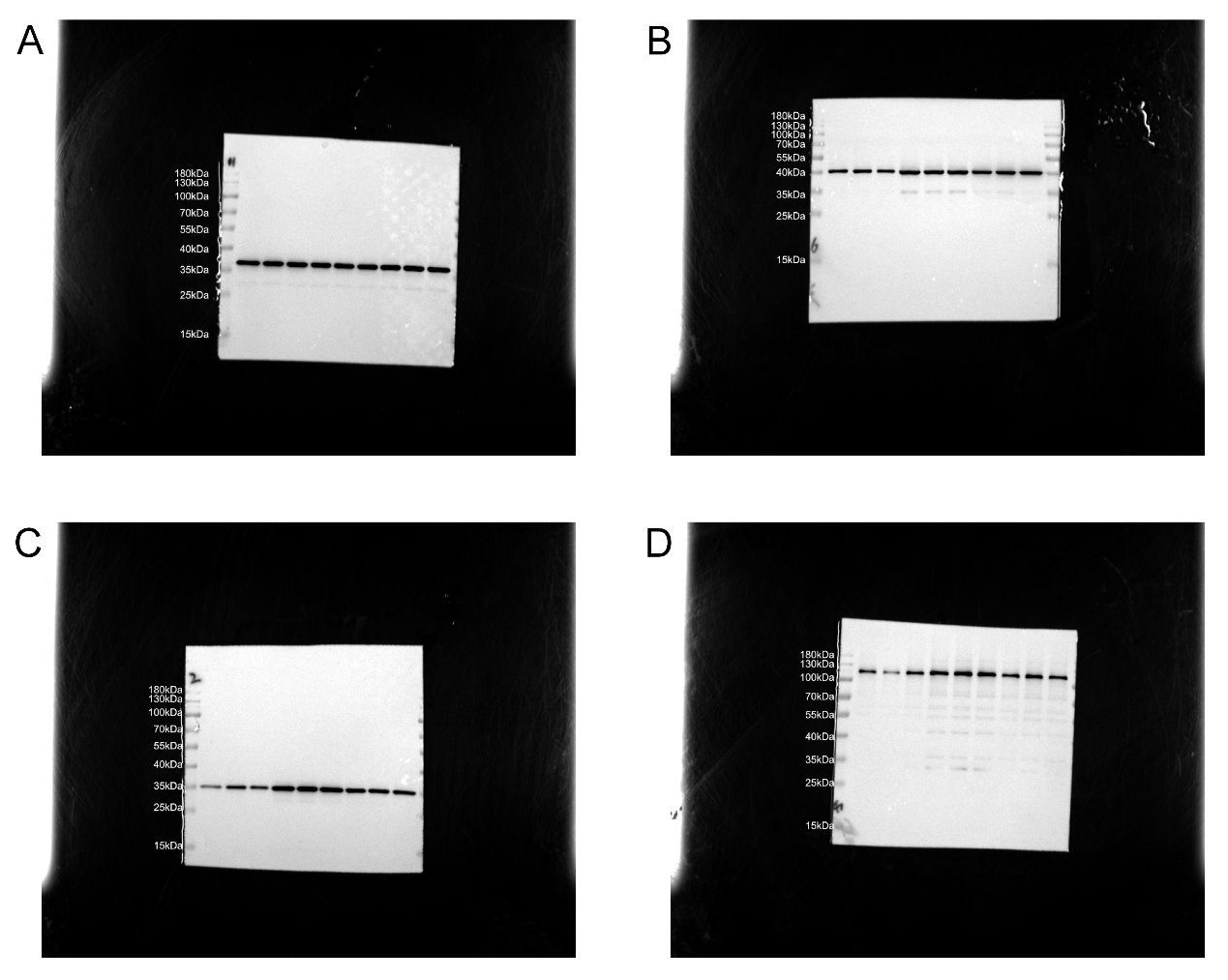


Table S1. Gene-specific primers used for qPCR.

| Genes | Forward | Reverse |
| --- | --- | --- |
| PGK1 | GTCGTGATGAGGGTGGACTTC | TAAGCACAACCGACTTGGCTC |
| LDHA | ATGAGCTTGCCCTTGTTGATGT | AAGATGTTCACGTTTCGCTGG |
| HIF1A | ACCGTGCCCCTACTATGTCG | GCCTTGTATGGGAGCATTAACTT |
| CD80 | CAGGTTCATTCATCTCTTTGTGC | GACAGCAATGCCTTTTCTCTCAC |
| CD86 | CAGTGTCTCCATCAGCCTATCTC | TTGTAGACGACCAGCAGAAAGAG |
| GAPDH | CTGGAGAAACCTGCCAAGTATG | GGTGGAAGAATGGGAGTTGCT |

Table S2. Active components and targets of herbs.

| Herbs | DAN SHEN | DANG GUI | SHA REN | TAN XIANG | HUANG QI |
| --- | --- | --- | --- | --- | --- |
| Components | Tanshinone IIA | Succinic Acid | Linalool | Isovitexin | quercetin |
|  | Succinic Acid | Phytolaccoside E | Alpha-Amyrin | Isoorientin | Neferine |
|  | Linalool | 1,4-NAPHTHOQUINONE | oleic acid | METHYL PALMITATE | METHYL PALMITATE |
|  | dehydrocostus lactone | Kainic Acid | Hyperoside | (3R,6E)-Nerolidol | kaempferol |
|  | Carnosol | Tetradecane | Humulene | ETHYL ACETATE | 7-O-methylisomucronulatol |
|  | Salvianolic Acid B | METHYL PALMITATE | METHYL PALMITATE | luteolin | formononetin |
|  | Danshensu | Senkyunolide A | (3R,6E)-Nerolidol | isorhamnetin | FA |
|  | METHYL PALMITATE | Butylphthalide | 3-Carene | oleic acid | isorhamnetin |
|  | Aucubin | Brefeldin A | (-)-Borneol | Toluene | Calycosin |
|  | cryptotanshinone | beta-sitosterol | L-Lactic Acid | Bergapten | Astragaloside A |
|  | (-)-Borneol | Stigmasterol | beta-sitosterol | OLEANOLIC ACID | Uridine |
|  | Isotanshinone II | 3-butylidene-phalide | Stigmasterol |  | daidzein |
|  | dan-shexinkum d | Uridine | (+)-Terpinen-4-Ol |  | rutin |
|  | neocryptotanshinone ii | Scopolin | Toluene |  | Ethinyl Estradiol |
|  | dihydrotanshinlactone | Isoimperatorin | Isoquercitrin |  | Isoquercitrin |
|  | miltionone Ⅰ | Phenol | Vanillic Acid |  | Vanillic Acid |
|  | deoxyneocryptotanshinone | (-)-Alpha-Terpineol | (+)-Catechin |  | Rhapontigenin |
|  | digallate | Dictamnine | Germacrone |  |  |
|  | 3,4-Dihydroxybenzaldehyde | Depinar | Acitretin |  |  |
|  | luteolin |  | CARVONE |  |  |
|  | Beta-Cryptoxanthin |  | (-)-Alpha-Terpineol |  |  |
|  | Isoimperatorin |  |  |  |  |
|  | neocryptotanshinone |  |  |  |  |
|  | tanshinone Ⅵ |  |  |  |  |
|  | Dehydrotanshinone II A |  |  |  |  |
|  | 2-isopropyl-8-methylphenanthrene-3,4-dione |  |  |  |  |
|  | 4-methylenemiltirone |  |  |  |  |
|  | Isocryptotanshinone |  |  |  |  |
| Targets | ABAT | ABCB1 | ABAT | AARS1 | ABAT |
|  | ABCA1 | ACE | ABCB1 | ABAT | ABCB1 |
|  | ABCB1 | ACHE | ABCC1 | ACHE | ABCC1 |
|  | ABCC1 | ADH1C | ABCC2 | ACMSD | ABCG2 |
|  | ABCG2 | ADORA1 | ABCG2 | ACP1 | ACACA |
|  | ACACA | ADORA2A | ABCG5 | ACP4 | ACE |
|  | ACHE | ADORA2B | ABCG8 | ADCY2 | ACHE |
|  | ACMSD | ADORA3 | ACHE | ADORA3 | ACP4 |
|  | ACP1 | ADRA1B | ACP4 | ADRB2 | ACPP |
|  | ACP4 | ADRA1D | ADH1C | ADTRP | ADH1B |
|  | ACTA1 | ADRA2A | ADORA3 | AKR1B1 | ADH1C |
|  | ADCY2 | ADRB1 | ADRA1A | AKR1B10 | ADORA1 |
|  | ADORA3 | ADRB2 | ADRA1B | AKT1 | ADORA2A |
|  | ADRA1A | AGTR2 | ADRA1D | ALOX12 | ADORA2B |
|  | ADRA1B | AIFM1 | ADRA2A | ALOX15 | ADORA3 |
|  | ADRA1D | AKR1B1 | ADRA2B | ALOX5 | ADRA1A |
|  | ADRA2A | AKR1B10 | ADRB1 | AMD1 | ADRA1B |
|  | ADRA2B | AKR1C2 | ADRB2 | AMY1A | ADRA1D |
|  | ADRA2C | AKR1C3 | ADRB3 | APEX1 | ADRA2C |
|  | ADRB2 | AKR1C4 | AHR | Api5-ps | ADRB1 |
|  | ADRB3 | AKR1D1 | AHRR | APP | ADRB2 |
|  | ADTRP | AKT1 | AHSA1 | AR | ADRB3 |
|  | AHSA1 | ALB | AKR1B1 | ATAD2B | ADTRP |
|  | AKR1B1 | ALDH5A1 | AKR1B10 | ATP2C1 | AHCYL1 |
|  | AKR1B10 | ALOX12 | AKT1 | BACE1 | AHR |
|  | Akr1c12 | ALOX15 | ALAS1 | BAX | AHSA1 |
|  | AKR1C2 | ALOX5 | ALK | BCAT1 | AKR1B1 |
|  | AKR1C3 | ANXA5 | ALOX12 | BCHE | AKR1B10 |
|  | AKR1C4 | AOX1 | ALOX15 | BCL2 | Akr1c12 |
|  | AKR1E2 | APEX1 | ALOX5 | BCL2L1 | AKR1C3 |
|  | AKT1 | APOE | ALOXE3 | BDNF | AKT1 |
|  | ALDH5A1 | APP | ALPI | BECN1 | ALAD |
|  | ALDH7A1 | ARF1 | ALPL | BHMT | ALB |
|  | ALK | ARFGEF3 | AMY1A | BHMT2 | ALOX12 |
|  | ALOX12 | ARHGAP1 | ANK2 | BIRC5 | ALOX15 |
|  | ALOX15 | ARHGDIA | ANKEF1 | CA1 | ALOX5 |
|  | ALOX5 | ATP5D | ANKHD1 | CA12 | ALOXE3 |
|  | ALOXE3 | ATP5F1A | ANKRA2 | CA2 | ALPI |
|  | ALPI | ATP5F1B | ANKRD1 | CA4 | ALPL |
|  | ALPL | AXL | ANKRD10 | CA7 | AMY1A |
|  | AMY1A | BACE1 | ANKRD16 | CALM3 | APEX1 |
|  | AOX1 | BAG3 | ANKRD37 | CAMK4 | Api5-ps |
|  | APEX1 | BAX | ANKS4B | CASP3 | APOB |
|  | Api5-ps | BCL2 | ANTXR2 | CASP7 | APP |
|  | APP | BCL2L1 | AOC2 | CASP9 | AR |
|  | Appbp2os | BCL2L11 | APEX1 | CAT | ARNTL2 |
|  | AQP1 | BCL2L2 | Api5-ps | CCL3 | ASNS |
|  | AQP3 | BDNF | APP | CCNA2 | ASRGL1 |
|  | AQP8 | BHMT | AQP4 | CCNB1 | ATP5D |
|  | AQP9 | BHMT2 | AR | CCND1 | ATP5F1B |
|  | AR | BIK | ARHGAP17 | CD38 | Atp5k-ps2 |
|  | ATAD2B | BIRC5 | ARL6IP5 | CD40LG | BACE1 |
|  | AURKB | C1QBP | ARNTL2 | CDC25B | BAX |
|  | AVPR2 | C5AR1 | ARRB1 | CDK2 | BCHE |
|  | AXL | CA1 | ASB2 | CDK4 | BCL2 |
|  | BACE1 | CA12 | ASB9 | CDK6 | BCL2L1 |
|  | BAX | CA13 | ASGR1 | CDKN1A | BDNF |
|  | BCAT1 | CA14 | ASIC1 | CEBPD | BHMT |
|  | BCHE | CA2 | AURKB | CHEK1 | BIRC5 |
|  | BCL2 | CA3 | AVPR2 | COQ3 | BLVRB |
|  | BCL2L1 | CA4 | AXL | COX10 | C5AR1 |
|  | BHMT | CA5A | BACE1 | CREB1 | CA1 |
|  | BHMT2 | CA5B | BAX | CSNK2A1 | CA12 |
|  | BIRC5 | CA6 | BCHE | CTH | CA2 |
|  | BMP2 | CA7 | BCL2 | CTNS | CA3 |
|  | CA1 | CA9 | BCL2A1 | CTSD | CA4 |
|  | CA12 | CACNA1C | BDNF | CTSL | CA7 |
|  | CA13 | CACNA1D | BMP2 | CXCL8 | CA9 |
|  | CA14 | CACNA1F | BTG1 | CYP19A1 | CALCA |
|  | CA2 | CACNA1H | CA1 | CYP1A1 | CALCB |
|  | CA3 | CACNA1S | CA12 | CYP1A2 | CALM3 |
|  | CA4 | CACNA2D1 | CA14 | CYP1B1 | CASP3 |
|  | CA5A | CACNA2D2 | CA2 | CYP2A6 | CASP8 |
|  | CA5B | CACNB2 | CA3 | CYP2C19 | CASP9 |
|  | CA6 | CALR | CA4 | CYP2C9 | CAT |
|  | CA7 | CASP3 | CA5A | CYP2D6 | CAV1 |
|  | CA9 | CASP8 | CA5B | CYP2E1 | CBR1 |
|  | CALCR | CASP9 | CA6 | CYP3A4 | CCL2 |
|  | CALM3 | CCL2 | CA7 | DAPK1 | CCNA2 |
|  | CAMK2B | CCL5 | CA9 | DGKD | CCNB1 |
|  | CASP3 | CCNE1 | CAMK1D | DHTKD1 | CCND1 |
|  | CASP7 | CCR5 | CAMK2B | DNTT | CD276 |
|  | CASP8 | CDK6 | CAMK4 | Dpml | CD40LG |
|  | CASP9 | CHRM1 | CASP3 | DPP4 | CDH1 |
|  | CCL20 | CHRM2 | CASP7 | E2F1 | CDK1 |
|  | CCL4 | CHRM3 | CASP8 | EGFR | CDK2 |
|  | CCNA2 | CHRM4 | CASP9 | EHMT2 | CDK6 |
|  | CCNB1 | CHRNA2 | CASR | ERBB2 | CDKN1A |
|  | CCND1 | COMP | CAT | ESR1 | CEBPA |
|  | Ccpg1os | CTBP1 | CCK | ESR2 | CHDH |
|  | CCR4 | CTSB | CCL2 | F3 | CHEK1 |
|  | CD1A | CTSL | CCL3 | F7 | CHEK2 |
|  | CD38 | CXCL1 | CCNB1 | FABP2 | CHRM1 |
|  | CD40 | CXCL2 | Ccpg1os | FABP3 | CHRM2 |
|  | CD40LG | CXCL8 | CCR4 | FABP4 | CHRM3 |
|  | CD83 | CXCR4 | CCR5 | FABP5 | CHRM4 |
|  | CD86 | CYCS | CD38 | FASN | CHRM5 |
|  | CDC25B | CYP19A1 | CD40LG | FLT3 | CHRNA7 |
|  | CDK1 | CYP1A1 | CD55 | FOLH1 | CHUK |
|  | CDK2 | CYP1A2 | CDK1 | FOXM1 | Ciaaq1 |
|  | CDK4 | CYP2A13 | CDK6 | GAA | CLDN4 |
|  | CDK6 | CYP2A6 | CDKN2B | GABRA1 | COL1A1 |
|  | CDKN1A | CYP2C19 | CDKN2C | GATM | COL3A1 |
|  | CDKN2A | CYP2C9 | CEBPA | GBA | COX10 |
|  | CES1 | CYP2D6 | CHRM1 | GFAP | Cox20b |
|  | CES2 | CYP3A4 | CHRM2 | GLB1 | CRP |
|  | CHEK1 | CYTB | CHRM3 | GLO1 | CRTC1 |
|  | CHRM1 | DAO | CHRM4 | GLRA1 | CSNK2A1 |
|  | CHRM2 | DGKD | CHRNA2 | GOT1 | CTSD |
|  | CHRM3 | DMP1 | Ciaaq1 | GPR35 | CXCL1 |
|  | CHRM4 | DPP4 | CITED1 | GPT | CXCL10 |
|  | CHRM5 | DRD1 | CNR2 | GRIA1 | CXCL11 |
|  | CHRNA2 | DUOX2 | COL1A1 | GRIA2 | CXCL2 |
|  | Ciaaq1 | DUSP3 | COQ3 | GRIA4 | CXCL3 |
|  | CNR1 | EDNRA | Cox20b | GRIK2 | CXCL8 |
|  | CNR2 | EEF1A1 | CPT1A | GRIK5 | CYP19A1 |
|  | COL1A1 | EEF1A2 | CPT1B | GRIN1 | CYP1A1 |
|  | COMT | EEF2 | CREB1 | GRIN3A | CYP1A2 |
|  | COX10 | EGFR | CREG1 | GRM1 | CYP1B1 |
|  | Cox20b | EGLN1 | CRP | GRM2 | CYP2C19 |
|  | CPT1B | EHMT2 | CTH | GRM3 | CYP2C9 |
|  | CREG1 | EIF4A1 | CTSD | GRM4 | CYP2D6 |
|  | CRP | ELAVL1 | CUTA | GRM5 | CYP3A4 |
|  | CTBP1 | ENO1 | CXCL8 | GRM8 | CYTB |
|  | CTH | EPHX2 | CXCR1 | GSK3B | DAPK1 |
|  | CTNS | ERBB2 | CXCR4 | GSTP1 | DHTKD1 |
|  | CTSD | ERN1 | CYP19A1 | HIF1A | DIO1 |
|  | CTSL | ESR1 | CYP1A1 | HMGCR | DPP4 |
|  | CXCR1 | ETFDH | CYP1A2 | HMOX1 | DRD1 |
|  | CYP19A1 | F10 | CYP1B1 | HSD17B1 | DUOX2 |
|  | CYP1A1 | F2 | CYP2A6 | HSD17B2 | DUSP3 |
|  | CYP1A2 | F3 | CYP2C19 | HSF1 | E2F1 |
|  | CYP1B1 | F7 | CYP2C8 | HSP90AA1 | E2F2 |
|  | CYP2B6 | F9 | CYP2C9 | HSPA1B | EBAG9 |
|  | CYP2C19 | FABP2 | CYP2D6 | HSPA4 | EGF |
|  | CYP2C8 | FABP3 | CYP2E1 | HSPB1 | EGFR |
|  | CYP2C9 | FABP4 | CYP2J2 | HSPB2 | EHMT2 |
|  | CYP2D6 | FABP5 | CYP3A4 | HTR7 | EIF6 |
|  | CYP2J2 | FGF2 | CYP4F3 | ICAM1 | ELK1 |
|  | CYP3A4 | FUS | DAPK1 | Idd6.3 | EPHX2 |
|  | DAPK1 | GAA | DBI | IDH1 | EPO |
|  | DHTKD1 | GABRA1 | DGKD | Ifi206 | ERBB2 |
|  | DIO1 | GABRR1 | DHFR | IFNG | ERBB3 |
|  | DNMT1 | GBA | DHTKD1 | IKBKB | ERG |
|  | Dpml | GBF1 | DIO1 | IL10 | ESR1 |
|  | DPP4 | GDF10 | DLD | IL2 | ESR2 |
|  | DRD1 | GDNF | DLGAP5 | IL4 | F3 |
|  | DRD2 | GGCX | DMP1 | IL6 | F7 |
|  | DRD5 | GLRA1 | Dpml | INSR | F8A1 |
|  | DUOX2 | GRIA1 | DPP4 | JUN | FABP3 |
|  | DUSP3 | GSK3B | DRD1 | KARS | FABP4 |
|  | EBAG9 | HCAR2 | E2F1 | KCNA1 | FCER2 |
|  | ECE1 | HCN4 | E2F2 | KCNA10 | FGFR2 |
|  | EDN1 | HDAC1 | E2F3 | KCNA2 | FLT3 |
|  | EDNRA | HDAC2 | E2F5 | KCNA3 | FOLH1 |
|  | EGFR | HDAC3 | EDNRA | KCNA4 | FOLR1 |
|  | Egfros | HDAC6 | EGFR | KCNA5 | FOLR2 |
|  | EGLN1 | HDAC8 | EGLN1 | KCNA6 | FOLR3 |
|  | EHMT2 | HIF1A | EGR3 | KCNA7 | FOS |
|  | EIF6 | HMGCR | EHMT2 | KCNB1 | GAA |
|  | ENPP7 | HMOX1 | EIF6 | KCNB2 | GABRA1 |
|  | EPHX2 | HNRNPA3 | ELOVL6 | KCNC1 | GAL |
|  | ERBB2 | HNRNPD | ENPP7 | KCNC2 | GATM |
|  | ESR1 | HNRNPF | EPHB1 | KCNC3 | GBA |
|  | ESR2 | HRH1 | EPHX2 | KCNC4 | GCG |
|  | ESRRA | HRH2 | ERBB2 | KCND1 | GGT5 |
|  | ESRRB | HRK | ERBB3 | KCND2 | GH1 |
|  | EZH2 | HSP90AA1 | ESPN | KCND3 | GJA1 |
|  | F2 | HSP90AB1 | ESR1 | KCNH2 | Gja6 |
|  | F3 | HSPA8 | EVI2A | KDM4E | GLUL |
|  | F7 | HTR2A | F2 | KMT2A | Gnf4 |
|  | F8A1 | HTR7 | F7 | MAOA | GRIA2 |
|  | FABP3 | HULC | FABP1 | MAOB | GSK3B |
|  | FABP4 | ICAM1 | FABP2 | MAP2 | Gssos2 |
|  | FASN | IDH1 | FABP3 | MAPK1 | GSTM1 |
|  | FFAR2 | IDO1 | FABP4 | MAPK14 | GSTM2 |
|  | FFAR3 | IFNG | FABP5 | MAPT | GSTP1 |
|  | FLT3 | IL10 | FASN | MCL1 | HAS2 |
|  | FOS | IL1B | FCGR2B | MDM2 | HIF1A |
|  | FUT1 | IL1RN | FEM1C | ME1 | HK2 |
|  | FYN | IL2 | FILIP1L | MET | HMOX1 |
|  | GAA | IL4 | FLT3 | METAP2 | HPSE |
|  | GABRA1 | IL6 | FOS | MGLL | HSD11B2 |
|  | GABRE | ITPKA | G6PD | Mipepos | HSD17B1 |
|  | GABRG3 | JUN | GAA | MMP1 | HSD17B2 |
|  | GAP43 | KCNH2 | GABPB2 | MMP12 | HSD3B1 |
|  | GATM | KDM4E | GABRA1 | MMP13 | HSD3B2 |
|  | GBA | KDR | GATM | MMP2 | HSF1 |
|  | GCG | KIF5B | GBA | MMP3 | HSP90AA1 |
|  | GCLC | KMT2A | GFAP | MMP9 | HSPA5 |
|  | GCLM | LDHA | GLB1 | Mod2 | HSPA8 |
|  | GJA1 | LIPG | GLO1 | MPO | HSPB1 |
|  | GLB1 | LTA4H | GNMT | MTOR | HTR2A |
|  | GLO1 | LTC4S | GPR3 | MTR | HTR3A |
|  | GOT1 | MAOA | GPR35 | MTRR | HTR7 |
|  | GPR35 | MAOB | GPT | NCF1 | ICAM1 |
|  | GPT | MAP1LC3B | GPT2 | NCOA1 | Idd6.3 |
|  | GRIN1 | MAP2 | GRIA2 | NCOA2 | IDH1 |
|  | GSK3B | MAP2K1 | GRIN1 | NEU1 | IFNG |
|  | Gssos2 | MAPK1 | GSK3B | NFE2L2 | IGF1R |
|  | GSTM1 | MAPK14 | GSN | NFKB1 | IGF2 |
|  | GSTP1 | MAPK3 | GSR | NFKBIA | IGFBP1 |
|  | HAS2 | MAPK8 | Gssos2 | NOS2 | IGFBP3 |
|  | HCAR2 | MAPK9 | GSTM1 | NOS3 | IKBKB |
|  | HDAC6 | MAPT | GSTP1 | NOSTRIN | IL10 |
|  | HERC5 | MB | HAMP | NQO1 | IL12B |
|  | HIF1A | MDM2 | HAS2 | NR1H4 | IL1A |
|  | HMOX1 | MET | HCN4 | NUF2 | IL1B |
|  | HRAS | METAP2 | HDAC6 | OLR1 | IL2 |
|  | HRH3 | MIF | HDC | OXA1L | IL4 |
|  | HSD11B1 | MIR203A | HERC5 | PARP1 | IL6 |
|  | HSD17B1 | MKI67 | HIF1A | PCCB | INSR |
|  | HSD17B2 | MMP1 | HIVEP2 | PCNA | IRAK4 |
|  | HSP90AA1 | MMP2 | HK2 | PDE5A | IRF1 |
|  | HTR1A | MMP3 | HMGCR | PIM1 | JUN |
|  | HTR1B | MMP9 | HMGCS1 | PKLR | KCNH2 |
|  | HTR2A | MPG | HMOX1 | PLA2G2A | KDM4E |
|  | HTR2C | MPO | HNF4G | PLB1 | KDR |
|  | HTR3A | MTR | HNRNPLL | PLG | KMT2A |
|  | HTR7 | MTRR | HSD11B1 | PM20D2 | KSR2 |
|  | HULC | NCF1 | HSD17B1 | POLB | LHCGR |
|  | ICAM1 | NCL | HSD17B2 | Polr2k-ps | LTF |
|  | Idd6.3 | NCOA1 | HSP90AA1 | PPARA | LYZ |
|  | IDH1 | NCOA2 | HSPA5 | PPARD | MAOA |
|  | Ifi206 | NDUFS3 | HTR1A | PPARG | MAOB |
|  | IFNG | NFE2L2 | HTR2A | PPIA | MAP2 |
|  | IGF1R | NFKB1 | HULC | PPIB | MAP3K7 |
|  | IGHG1 | NGF | IDH1 | Ppp1r18os | MAPK1 |
|  | IKBKB | NLN | IDI1 | PRSS1 | MAPK14 |
|  | IL10 | NME1 | IFIT1 | PTEN | MAPK3 |
|  | IL12A | NOS2 | IFIT2 | PTGES | Mapk6-ps1 |
|  | IL12B | NPY | IFNG | PTGS1 | MAPK8 |
|  | IL1A | NQO1 | IFNGR2 | PTGS2 | MAPT |
|  | IL2 | NR1H2 | IGF1R | PTPN1 | MET |
|  | IL4 | NR1I2 | IGF2 | PTPN11 | MGAM |
|  | IL6 | NR3C2 | IGHG1 | PTPN2 | MMP1 |
|  | INPPL1 | NRCAM | IL10 | PTPN6 | MMP2 |
|  | INS | NTF3 | IL1B | PTPRF | MMP3 |
|  | INSR | ODC1 | IL1R2 | PTPRS | MMP9 |
|  | IQGAP2 | OPRK1 | IL2 | PYGM | Mod2 |
|  | ITGB3 | OPRM1 | IL4 | RB1 | MPO |
|  | ITGB8 | PCNA | IL6 | RELA | MT-ND6 |
|  | ITK | PECAM1 | INS | RORC | MTOR |
|  | JUN | PENK | INSR | RTP1 | MYC |
|  | KARS | PGK1 | IRAK4 | SARDH | NARS |
|  | KCNH2 | PGR | ITGA6 | SERPIND1 | NARS2 |
|  | KDM4E | PHB | JUN | SIGMAR1 | NCF1 |
|  | KDR | PKD1 | KANK1 | SLC1A1 | NCOA1 |
|  | KEAP1 | PKD2 | KANK4 | SLC2A4 | NCOA2 |
|  | KMT2A | PLA2G1B | KCNH2 | SLC6A2 | NDUFS3 |
|  | KSR2 | PLAU | KDM4E | SLC6A3 | NFE2L2 |
|  | LAMP1 | PNP | KDR | SLC7A1 | NFKB1 |
|  | MAP2K1 | POLB | KIF23 | SLC7A11 | NFKBIA |
|  | MAPK1 | PON1 | KLF7 | SLC7A2 | NKX3-1 |
|  | MAPK11 | POR | KMT2A | SLC7A3 | NLRP3 |
|  | MAPK14 | PPARA | LCT | SLC7A4 | NOS2 |
|  | Mapk6-ps1 | PPARD | LPL | SOAT1 | NOS3 |
|  | MAPK8 | PPARG | Lprq3 | SOD2 | NOSTRIN |
|  | MAPKAPK2 | PPP2R1A | LTA4H | SRD5A2 | NQO1 |
|  | MAPT | PRKAB1 | MAOA | SYK | NR1I2 |
|  | MCL1 | PRKAG2 | MAOB | TARS | NR1I3 |
|  | MDM2 | PRKCA | MAP1S | TARS2 | Oaz2-ps |
|  | MET | PRKCE | MAP2 | TAS2R31 | ODC1 |
|  | METAP2 | PROC | MAP2K1 | TBXAS1 | OLR1 |
|  | MGAM | PROS1 | MAP3K8 | TCEAL7 | OPRD1 |
|  | MGLL | PRPS1 | MAPK1 | TERT | OPRM1 |
|  | Mipepos | PSMA1 | MAPK14 | THRB | OXA1L |
|  | MMP1 | PSMD11 | MAPK3 | TLR2 | P4HB |
|  | MMP12 | PTGS1 | Mapk6-ps1 | TLR4 | PARP1 |
|  | MMP13 | PTGS2 | MAPKAPK2 | TNF | PCOLCE |
|  | MMP2 | PTPN1 | MAPT | TNKS | PCYT1A |
|  | MMP3 | RAPGEF3 | ME1 | TNKS2 | PCYT1B |
|  | MMP9 | RELA | MET | TOP1 | PDE3A |
|  | Mod2 | RHOA | MGAM | TOP2A | PDE5A |
|  | MPG | RIPK1 | Mipepos | TOP2B | PGR |
|  | MPO | RIPK3 | MMP12 | TP53 | PIM1 |
|  | MTOR | RORC | MMP13 | TP53COR1 | PLAT |
|  | MTR | RPSA | MMP2 | TRPV1 | PLAU |
|  | MTRR | RXRA | MMP3 | TTR | PLG |
|  | MYC | RYR1 | MMP8 | TYR | PM20D2 |
|  | NCOA1 | SCN10A | MMP9 | UGT1A1 | PNP |
|  | NCOA2 | SCN5A | Mod2 | VARS | POLB |
|  | NCOA3 | SF3B3 | MOGAT2 | VEGFC | POLD1 |
|  | NEK2 | SFN | MPG | VEZF1 | POMC |
|  | NEK6 | SIGMAR1 | MPO | XDH | PON1 |
|  | NFE2L2 | SIRT1 | MTOR | XIAP | POR |
|  | NFKB1 | SLC22A1 | MXD1 |  | PPARA |
|  | NFKBIA | SLC28A1 | NCF2 |  | PPARD |
|  | NKD2 | SLC28A2 | NCOA1 |  | PPARG |
|  | NLRP3 | SLC28A3 | NCOA2 |  | PPARGC1A |
|  | NOS2 | SLC47A1 | NDUFS1 |  | PPIA |
|  | NOS3 | SLC5A11 | NEDD9 |  | PPIB |
|  | NOSTRIN | SLC6A2 | NEK2 |  | Ppp1r18os |
|  | NPM1 | SLC6A3 | NEK6 |  | PRKAA1 |
|  | NPY | SLC6A4 | NFE2L2 |  | PRKAG2 |
|  | NQO1 | SLCO1B1 | NFIB |  | PRKCA |
|  | NR1H2 | SLCO1B3 | NFKB1 |  | PRKCB |
|  | NR1H4 | SLCO2B1 | NFKB2 |  | PRSS1 |
|  | NR1I2 | SMAD2 | NFKBIA |  | PSG1 |
|  | NR3C1 | SNAI1 | NOS2 |  | PSMD3 |
|  | NR3C2 | SOAT1 | NOS3 |  | PTGER3 |
|  | NUAK1 | SRD5A1 | NOSTRIN |  | PTGER4 |
|  | NUF2 | SRD5A2 | NPY |  | Ptges3-ps |
|  | Oaz2-ps | SRD5A3 | NQO1 |  | PTGR1 |
|  | ODC1 | STAT3 | NR1H2 |  | PTGS1 |
|  | OPCML | STK11 | NR2C2 |  | PTGS2 |
|  | OPRD1 | SUCNR1 | NR3C2 |  | PTPN1 |
|  | OPRK1 | TERT | NUAK1 |  | PYGM |
|  | OPRM1 | TGFB1 | NUP62 |  | QRICH2 |
|  | OXA1L | THRB | Oaz2-ps |  | RASA1 |
|  | P2RX7 | TLR2 | OPRD1 |  | RASSF1 |
|  | P2RY12 | TLR8 | OPRK1 |  | RB1 |
|  | PARG | TNF | OPRM1 |  | RBP4 |
|  | PARP1 | TOMM22 | OR51E2 |  | RELA |
|  | PARP4 | TOMM70A | OXA1L |  | REN |
|  | PCCB | TP53 | OXNAD1 |  | RFK |
|  | PCNA | TPT1 | P4HB |  | RIT1 |
|  | PDE3A | TRAF6 | PDE5A |  | RORC |
|  | PDE5A | TRPA1 | PDX1 |  | RPS6KB1 |
|  | PGD | TRPM7 | PGD |  | RTP1 |
|  | PGR | TRPV1 | PGR |  | RUNX1T1 |
|  | PIK3CA | TRPV3 | PIGK |  | RUNX2 |
|  | PIK3CB | TTR | PIK3CG |  | RXRA |
|  | PIK3CD | TUFM | PIM1 |  | RXRB |
|  | PIK3CG | TYMP | PKN1 |  | S100A4 |
|  | PIM1 | TYR | PLA2G2A |  | SCN5A |
|  | PKN1 | UGT1A1 | PLA2G2D |  | SELE |
|  | PLA2G2D | UGT1A9 | PLAU |  | SERPIND1 |
|  | PLG | UNG | PLG |  | SERPINE1 |
|  | PLK1 | URAD | PLK1 |  | SFTPA1 |
|  | PM20D2 | VEGFA | PM20D2 |  | SI |
|  | POLB | WAS | PMAIP1 |  | SIRT1 |
|  | POLD1 | XDH | POLB |  | SLC19A1 |
|  | PPARA | YBX1 | POLD1 |  | SLC1A5 |
|  | PPARD | YWHAZ | PON1 |  | SLC28A1 |
|  | PPARG | ZEB1 | POR |  | SLC28A2 |
|  | PPIA |  | PPARA |  | SLC28A3 |
|  | PPIB |  | PPARD |  | SLC2A4 |
|  | Ppp1r18os |  | PPARG |  | SLC38A1 |
|  | PRKCA |  | PPP1R16A |  | SLC38A2 |
|  | PRKCB |  | PRKAA1 |  | SLC38A3 |
|  | PRSS1 |  | PRKCA |  | SLC38A4 |
|  | PSMD3 |  | PRKCB |  | SLC46A1 |
|  | PTEN |  | PRRT2 |  | SLC5A1 |
|  | PTGES |  | PSMC3IP |  | SLC5A12 |
|  | Ptges3-ps |  | PTEN |  | SLC5A2 |
|  | PTGS1 |  | PTGER3 |  | SLC5A5 |
|  | PTGS2 |  | Ptges3-ps |  | SLC5A6 |
|  | PTK2 |  | PTGS1 |  | SLC5A7 |
|  | PTPN1 |  | PTGS2 |  | SLC5A8 |
|  | PTPN11 |  | PTK2 |  | SLC6A2 |
|  | PTPN2 |  | PTPN1 |  | SLC6A3 |
|  | PTPN6 |  | PYGL |  | SLC6A4 |
|  | PTPRF |  | RAB1B |  | SLCO1B1 |
|  | PTPRS |  | RAB8B |  | SLCO1B3 |
|  | PYGL |  | RAD51 |  | SLCO2B1 |
|  | RARA |  | RAI14 |  | SLPI |
|  | RB1 |  | RARA |  | SOAT1 |
|  | RELA |  | RARB |  | SOD1 |
|  | RIPK1 |  | RARG |  | Sod1m |
|  | RORC |  | RASA1 |  | SOX2 |
|  | RPS6KB1 |  | RASSF1 |  | SPP1 |
|  | RRAGB |  | RB1 |  | STAT1 |
|  | RTP1 |  | RBP1 |  | STAT3 |
|  | RUNX1 |  | RELA |  | SULT1E1 |
|  | RXRA |  | RELB |  | SYK |
|  | SARDH |  | RGS1 |  | TCEAL7 |
|  | SCN5A |  | RIPK4 |  | TERT |
|  | SELP |  | RNASEL |  | TFAP2A |
|  | SERPIND1 |  | RNF149 |  | TGFB1 |
|  | SHBG |  | RORC |  | TH |
|  | SIRT1 |  | RPL27A |  | THBD |
|  | SKP2 |  | RRM2B |  | THRB |
|  | SLC2A4 |  | RTP1 |  | TIMP1 |
|  | SLC5A1 |  | RXRA |  | TLR4 |
|  | SLC5A2 |  | RXRB |  | TLR8 |
|  | SLC6A2 |  | RXRG |  | TNF |
|  | SLC6A3 |  | S1PR1 |  | TNKS |
|  | SLC6A4 |  | SARDH |  | TNKS2 |
|  | SLC7A1 |  | SCN4A |  | TOMM22 |
|  | SLC7A11 |  | SCN5A |  | TOMM70A |
|  | SLC7A2 |  | SCN9A |  | TOP1 |
|  | SLC7A3 |  | SDC2 |  | TOP2A |
|  | SLC7A4 |  | SERPINE1 |  | TOP2B |
|  | SLCO1B1 |  | SESN2 |  | Topbp1-ps1 |
|  | SLCO1B3 |  | SIGMAR1 |  | TP53 |
|  | SLCO2B1 |  | SIRT1 |  | TP53COR1 |
|  | SNCA |  | SKI |  | TRPM8 |
|  | SOAT1 |  | SLC12A4 |  | TRPV1 |
|  | SOD1 |  | SLC22A1 |  | TRPV2 |
|  | Sod1m |  | SLC22A5 |  | TRPV4 |
|  | SOD2 |  | SLC2A2 |  | TRPV5 |
|  | SRC |  | SLC38A2 |  | TRPV6 |
|  | STAT3 |  | SLC6A2 |  | Tstap198-15 |
|  | SUCNR1 |  | SLC6A3 |  | TTR |
|  | SYK |  | SLC6A4 |  | TYMP |
|  | TARS |  | SLC7A11 |  | TYR |
|  | TARS2 |  | SLC7A9 |  | TYRP1 |
|  | TBXAS1 |  | SLCO1B1 |  | UBE2N |
|  | TCEAL7 |  | SLCO1B3 |  | UGT1A1 |
|  | TERT |  | SLCO2B1 |  | USP5 |
|  | TGFB1 |  | SOAT1 |  | VCAM1 |
|  | THRB |  | SOD1 |  | VEGFA |
|  | TJP1 |  | Sod1m |  | VEGFC |
|  | TLR4 |  | SOD2 |  | VEZF1 |
|  | TNF |  | SOD3 |  | XDH |
|  | TNFRSF9 |  | SP1 |  |  |
|  | TNKS |  | SPHK1 |  |  |
|  | TNKS2 |  | SPP1 |  |  |
|  | TNNI3 |  | SRC |  |  |
|  | TOP1 |  | SRD5A2 |  |  |
|  | TOP2A |  | STAT3 |  |  |
|  | TOP2B |  | STX11 |  |  |
|  | Topbp1-ps1 |  | SYK |  |  |
|  | TP53 |  | TANGO6 |  |  |
|  | TP53COR1 |  | TEP1 |  |  |
|  | TRPM8 |  | TGFB1 |  |  |
|  | TTR |  | THRB |  |  |
|  | TYR |  | TJP1 |  |  |
|  | UGT1A1 |  | TLR2 |  |  |
|  | VARS |  | TLR4 |  |  |
|  | VCAM1 |  | TNF |  |  |
|  | VEGFA |  | TNFAIP3 |  |  |
|  | VEGFC |  | TNKS |  |  |
|  | VEZF1 |  | TONSL |  |  |
|  | XDH |  | TOP2A |  |  |
|  | XIAP |  | TP53 |  |  |
|  | ZEB1 |  | TP53COR1 |  |  |
|  |  |  | TRAPPC2 |  |  |
|  |  |  | TRAPPC6A |  |  |
|  |  |  | TRPA1 |  |  |
|  |  |  | TRPM7 |  |  |
|  |  |  | TRPM8 |  |  |
|  |  |  | TRPV1 |  |  |
|  |  |  | TRPV3 |  |  |
|  |  |  | TYR |  |  |
|  |  |  | TYRP1 |  |  |
|  |  |  | UCP2 |  |  |
|  |  |  | UGT1A1 |  |  |
|  |  |  | UGT1A6 |  |  |
|  |  |  | UGT1A9 |  |  |
|  |  |  | UGT2B4 |  |  |
|  |  |  | USP18 |  |  |
|  |  |  | VCAM1 |  |  |
|  |  |  | VEGFA |  |  |
|  |  |  | VN1R1 |  |  |
|  |  |  | XBP1 |  |  |
|  |  |  | XDH |  |  |
|  |  |  | XPO1 |  |  |
|  |  |  | ZC3HAV1 |  |  |
|  |  |  | ZCCHC2 |  |  |
